# Group-Based Interventions for Posttraumatic Stress Disorder: A Systematic Review and Meta-Analysis of the Role of Trauma Type

**DOI:** 10.1177/18911803261471160

**Published:** 2026-07-30

**Authors:** Éadaoin Whelan, Siobhán M. Griffin, Elayne Ahern, Alžběta Lebedová, Grace McMahon, Daragh Bradshaw, Orla T. Muldoon

**Affiliations:** 1 Centre for Social Issues Research, Department of Psychology, 8808 University of Limerick, Limerick, Ireland; 2 School of Psychology, Queens University Belfast, Belfast, UK

**Keywords:** group-based interventions, posttraumatic stress disorder, social identity, trauma

## Abstract

**Background:**

Post-traumatic stress disorder (PTSD) is associated with poor health outcomes. While group-based therapies offer accessible treatment options, their effectiveness remains unclear. Social identity factors, such as shared group membership and stigma, may influence treatment success.

**Objectives:**

This review examined: (1) the effectiveness of group-based treatments for PTSD symptoms, (2) differences between group and individual therapies, (3) whether trauma type (e.g., interpersonal, stigmatized) and group fit (e.g., gender, shared trauma) moderate outcomes, and (4) the role of group and social identity factors in existing research.

**Search Methods:**

A systematic search (April 2023, re-run May 2025) identified quantitative studies of group therapies (≥3 participants, ≥5 sessions) addressing PTSD symptoms, compared to individual or control conditions. Forty studies met criteria (23 RCTs, 17 non-RCTs) from an initial pool of 8,256 records. Interventions included group CBT, counselling, psychodynamic, and other approaches.

**Selection Criteria:**

A variety of group-based treatments were eligible for inclusion, such as group-based CBT, CPT, and alternative group treatments. In line with previous research, protocols had to include a minimum of three individuals per group and meet for at least five sessions. Interventions assessed PTSD symptoms pre-intervention and post-intervention using a valid and reliable measure.

**Data Collection and Analysis:**

Primary outcome data was collected using valid measures of PTSD (e.g. CAPS, PCL). Effect sizes were computed for outcomes within each study. Where a study provided more than one effect size for each outcome, we accounted for this in statistical analyses.

**Main Results:**

Across 40 studies in 11 countries, most involving women or mixed-gender groups, trauma types varied, with combat-related PTSD most common (*n* = 15). Overall, group-based interventions were significantly more effective than comparator conditions, producing moderate symptom reductions (*k* = 32; *g* = 0.63; 95% *CI* [0.36, 0.90]; *p* < .001). Effects were large and clinically meaningful compared to controls (minimal attention and waitlist controls). However, effects were weaker and non-significant compared to treatment-as-usual and active controls (same treatment in individual format). Group CBT, group psychotherapy, and alternative approaches (e.g., mindfulness-based therapy, music therapy) showed stronger effects. Older participants, larger groups (>8 participants), women-only groups, and groups with shared or distinct trauma types demonstrated the largest effect sizes. We planned to examine how group-based processes (e.g., belonging, cohesion, trust) influenced PTSD symptoms, but could not do so because only two studies explicitly measured these group-based factors.

**Authors’ Conclusions:**

Group-based interventions reduce PTSD symptoms. Larger effects were observed for certain group characteristics, such as participant age, group size, gender, and shared experiences; however, findings are exploratory and should be interpreted with caution. Some limitations should also be considered. The majority of included studies had some concerns or were at moderate risk of bias, there was unexplained heterogeneity between studies, as well as evidence of possible publication bias. However, sensitivity analyses excluding studies at high or serious risk of bias yielded similar findings. Future research should build on existing evidence to examine how social identity processes influence the effectiveness of group-based interventions for PTSD across diverse populations and formats.

## Plain Language Summary

**The Review in Brief:** Group-based therapies aim to support individuals, including those diagnosed with Post-Traumatic Stress Disorder (PTSD), and reduce negative outcomes. Findings indicate that group-based interventions were significantly more effective than comparison conditions and produced a moderate effect size on PTSD symptom reduction. However, more interventions are needed that specifically measure group-based social identity factors.

**What Is the Review About?** PTSD is associated with negative outcomes such as co-morbid psychiatric conditions, lower quality of life, and increased risk of suicide. While individual approaches to therapy offer some evidence of improvement, research findings involving group-based approaches are conflicted and less well understood. Group processes such as identification, shared experiences, and stigma may be influencing the results. This review synthesised existing evidence on the effectiveness of group-based interventions for PTSD.

**What is the Aim of This Review?** This Campbell systematic review examines the efficacy of group-based treatments for individuals diagnosed with PTSD and explore any group-based social identity factors that might influence results.

**What Are the Main Findings of This Review?** In total 40 studies from 11 countries, inclusive of 8,504 participants worldwide were included in this review. The weighted mean age of participants across studies was 43.3 years. Most studies were conducted with women only (*n* = 16) or mixed (*n* = 16) groups, only five studies were conducted with men, and three studies did not report on the gender composition of their groups. The mean number of participants per group-based treatment was ten.

15 studies focused on interventions with combat-related PTSD, followed by abuse, both physical and sexual (*n* = 6), a combination of different traumatic experiences (*n* = 6; studies where participants experienced a range of different traumas), multiple traumatic experiences (*n* = 5; studies where participants experienced repeated or numerous traumas), motor vehicle accidents (*n* = 2), PTSD related to a cancer diagnosis (*n* = 1), an earthquake (*n* = 1), a car-hijacking (*n* = 1), refugees (*n* = 1), and experienced a birth-related trauma as a mother (*n* = 1).

We found that overall, group-based interventions were significantly more effective than comparison conditions and produced a moderate effect size on PTSD symptom reduction.

There were differences in comparator type with comparisons against control groups (i.e. minimal attention and waitlist controls) indicating significant differences whereas comparisons against TAU and active controls were not statistically significant.

Group interventions were effective for both intentional (e.g. assault) and unintentional traumas (e.g. natural disasters). Groups with shared or different traumas showed larger effects than those with multiple traumas.

There was a small effect of age with greater reduction of symptoms amongst older participants.

There were differences in the type of group-based treatment with group psychotherapy showing the largest effect, followed by alternative therapies (e.g. mindfulness-based therapy), and group CBT. Other treatment types (e.g., movement-based, group cognitive processing therapy, and other group treatments) were not statistically significant, relative to the comparison conditions.

While 24 studies referred to social-identity or group-based factors only 2 studies explicitly measured them.

**What do the Main Findings of This Review Mean?** These findings have practical and theoretical implications. Findings demonstrated that group-based interventions are effective at reducing PTSD symptomology in individuals diagnosed with PTSD compared to control groups (minimal attention and waitlist controls). Effects were smaller for treatment-as-usual and active controls, but these differences were non-significant. Some limitations should also be considered. Many of the included studies had methodological limitations and were rated as moderate risk of bias or as having some concerns, according to heterogeneity tests, findings also varied between studies, and there was some evidence of publication bias. However, when studies at high or serious risk of bias were excluded, the overall findings remained similar. Consequently, findings need to be interpreted in the context of trauma type and group characteristics. Specifically, while findings highlight the importance of group-based approaches, they also emphasise group-based factors such as participant age, group size, shared experiences, and gender may be linked to larger effects. Additionally, findings also highlight a reliance on US-based, military focused studies. Future studies should aim to target more diverse populations and contexts. Finally, while social identity and group-based mechanisms are hypothesised to influence the effect of treatments, they remain underexplored in the literature.

**How up to Date Is This Review?** Authors searched for studies published up to May 2025.

## Background

### The Problem, Condition or Issue

Incidence of posttraumatic stress disorder (PTSD) varies across populations, countries, and trauma types (Kessler et al., 2017; Liu et al., 2017), with rough estimates suggesting about 3.5% of the population experience PTSD at some point in their lifetime (Kessler et al., 2005). Often PTSD is a chronic and life debilitating condition (Hidalgo & Davidson, 2000). It involves four clusters of symptoms relating to: re-experiencing symptoms of a distressing event (e.g., intrusive thoughts), hyperarousal (e.g., irritability, increased startle response), avoidance of any reminders of the distressing event, and numbing and/or negative moods or cognitions associated with the event, as defined by the fifth edition of the Diagnostic and Statistical Manual of Mental Disorders (DSM-5; American Psychiatric Association, 2013). Symptoms of PTSD also known as posttraumatic stress have been reported after a variety of traumatic and distressing events including, but not limited to, sexual violence (Amone-P’olak et al., 2018), childhood abuse (Barlow et al., 2017), military combat and deployment (Armenta et al., 2019; Campbell et al., 2019), the COVID-19 pandemic (Tyra et al., 2021; Xiong et al., 2020), motor vehicle accidents (Blanchard et al., 1995), exposure to violence (Cuartas & Roy, 2019; Trevillion et al., 2012), mass shootings (Bardeen et al., 2013), and natural disasters (An et al., 2019; Muldoon et al., 2017).

The experience of PTSD has far reaching consequences. People who experience PTSD often experience co-morbid psychiatric conditions (Pietrzak et al., 2011), report a lower quality of life and poorer functional outcomes (Magruder et al., 2004; Schnurr et al., 2006; Zatzick, 2002), and are at increased risk for suicide (Guerra & Calhoun, 2011; Sareen et al., 2007). Furthermore, PTSD incurs a high economic burden, both in terms of direct and indirect costs (Dams et al., 2020; McGowan, 2019; von der Warth et al., 2020).

Effective means to treat PTSD remains an important challenge; treating PTSD optimally can help improve well-being and reduce the economic burden on society by reducing associated indirect costs associated with impaired functioning at work and in social domains (e.g., absenteeism). However, PTSD treatment guidelines differ in their consensus of what treatments are efficacious, particularly the utility of group treatments for treating PTSD. For instance, some guidelines do not address group interventions (American Psychological American Psychiatric Association, 2013; National Institute for Health and Care Excellence (NICE), 2018), while other guidelines provide recommendations for certain types of group therapy. As an example, the International Society for Traumatic Stress Studies guidelines (International Society for Traumatic Stress Studies [ISTSS], 2018) state that trauma-focused group cognitive behaviour therapy (CBT) is effective but there is insufficient evidence to make recommendations for other types of group treatments (e.g., group interpersonal therapy). Indeed, a meta-analysis by Lewis and colleagues (2020), which informed the current ISTSS recommendations, showed that group-based treatments with a trauma focus reduced symptoms (compared to waitlist controls and/or care as usual conditions) but evidence was mixed for group therapies that were not trauma focused (e.g., group relaxation, psychoeducation, psychodynamic therapy). Although the Department of Veterans Affairs and Department of Defense (VA/DoD) (2017) provides a moderate recommendation for group therapy, these guidelines do not specify what type of group therapy. Further, attention to the group processes that might foster therapeutic effects is lacking.

It appears from these guidelines that CBT-based group therapies are an effective way of treating PTSD, but it is unclear what other types of group treatments for PTSD are of value, for who, and what group processes are involved. It is also worth noting that these recommendations primarily stem from the results of a handful of randomized controlled trials (RCTs; Hamblen et al., 2019). A key aspect, overlooked by research to date, is attention to the role of shared group membership and the associated group-based social identity processes that groups can offer; a limitation that this review and meta-analysis will address.

The social identity approach to health demonstrates that shared group memberships (e.g., belonging to a support group, same gender identity group, group membership shaped by a shared type of trauma) can affect psychological health, both positively (Cruwys, Haslam, et al., 2014) and negatively (Kellezi & Reicher, 2012). Shared group memberships can offer people psychological resources through processes such as social identification with others in the group, group cohesion, perceived fit with others, and/or a sense of common fate (Borek et al., 2019; Steffens et al., 2021). The psychological resources offered through groups (e.g., belonging, social support, felt understanding) may help buffer the effects of trauma and alleviate PTSD symptoms. However, at times shared group membership might serve as a social curse (e.g., if it is a stigmatized identity) and this can worsen psychological outcomes, such as PTSD, as it can undermine belonging with the group (e.g., Muldoon, Walsh, et al., 2019). These processes remained underexplored as potential mechanisms through which group therapies may have implications for PTSD treatment. The proposed review will therefore synthesize existing evidence, including both RCTs and non-RCTs, and will investigate factors that may contribute to the efficacy of group-based treatments.

### The Intervention

Group-based interventions can offer an effective way to reduce PTSD symptoms (J. G. Beck & Coffey, 2005; Sloan et al., 2013). Existing interventions for PTSD, such as CBT (J. G. Beck et al., 2009), dialectical behaviour therapy (Bradley & Follingstad, 2003), psychotherapy (Classen et al., 2011), and cognitive processing therapy (Chard, 2005) are often conducted in group format and can effectively reduce PTSD symptoms. Although the type of treatment taking place within these groups is often the sole focus of the researchers’ investigations, evidence has shown that the provision of psychological treatments for PTSD in a group format can alleviate PTSD symptoms to a greater degree compared to waitlist controls and/or care as usual conditions (for meta-analyses see; Barrera et al., 2013; Schwartze et al., 2019; Sloan et al., 2013). While individual and group-based therapies may include similar content and psychological approaches, groups offer further potential by harnessing the benefits of the social group. Furthermore, economically, provision of psychological treatments in a group format are more cost and time effective than individual-level treatment (from a clinician standpoint) as multiple people can be treated at once by either one or two facilitators who lead the sessions (e.g., J. G. Beck & Coffey, 2005).

### How the Intervention Might Work

Regardless of the specific therapeutic approach used in a group treatment, we argue that it is the group-based nature of the treatment that is most beneficial, and this may offer advantages over individual-level treatments. This hypothesis stems from the social identity approach to health which emphasizes the importance of group memberships for everyday life (Tajfel & Turner, 1979; Turner et al., 1987). Being a member of a group is thought to be beneficial for psychological well-being and health (Cruwys, Alexander Haslam, et al., 2014; C. Haslam et al., 2008; Jetten et al., 2014) as groups can provide individuals with social, psychological, and material resources to cope with the adverse effects of life change, including trauma (C. Haslam et al., 2018; Jones et al., 2012; Kearns et al., 2018; Muldoon, Haslam, et al., 2019; Walsh et al., 2015).

For instance, belonging to a social group has been shown to be associated with lower risk of depression (Cruwys et al., 2013; Sani et al., 2012; Seymour-Smith et al., 2017), greater well-being (Iyer et al., 2009; Sani et al., 2015), and lower PTSD symptoms (Jones et al., 2012; Muldoon & Downes, 2007). As such, group-based psychological treatments for PTSD may offer additional benefits for well-being through the provision of psychological resources, such as a sense of belonging and social support (Avanzi et al., 2018; C. Haslam et al., 2016; S. A. Haslam et al., 2005; Walter et al., 2016).

The provision of social support is particularly important for people who have experienced trauma, as often the experience of trauma leads to social withdrawal (Hofmann et al., 2003) and sometimes results in the loss of valued group memberships and identity loss (Muldoon, Haslam, et al., 2019). Further, trauma can influence a person’s ability to engage with and bond with others (Charuvastra & Cloitre, 2008), serving to amplify the isolation experienced. This is particularly evident in traumas that are stigmatized in society (Kellezi & Reicher, 2012). Social isolation can in turn affect the severity of PTSD symptoms as a lack of social support can impair a person’s ability to regulate their distress, leading to poorer clinical outcomes (Price et al., 2018). Group-based treatments may help provide valuable social support for individuals who may be facing isolation as a result of their trauma and ultimately improve clinical outcomes.

Moreover, group-based treatments can put people in contact with others ‘like them’ (i.e., others who have also experienced a similar trauma). Being part of a group may foster social identification with the group (i.e., the degree to which a person feels the group positively informs their definition of self; Postmes et al., 2013). Greater social identification with the group provides resources such as acceptance, perceived efficacy, support, and solidarity (Cruwys & Gunaseelan, 2016), in an effect known as the social cure (Jetten et al., 2012). Importantly, identification with others in the group is believed to help mitigate the effects of trauma and reduce PTSD symptoms (Muldoon, Haslam, et al., 2019).

However, it is worth noting that group-based treatments might not always be beneficial. Some researchers have raised concerns that hearing about another person’s trauma might be retraumatising for some, or that disclosing traumatic experiences in a group setting might lead to unhelpful social comparisons, such as feeling that one’s trauma is not as legitimate as other group members (J. G. Beck & Coffey, 2005; Courtois & Ford, 2012). Similarly, reliving or confronting traumatic memories in a group format might be uncomfortable for some people (Røberg et al., 2018). This has implications for the efficacy of group-based treatments as confronting traumatic memories is thought to be a key process in treating PTSD symptoms. In this regard, it may be the case that individual-based therapies might show advantages over group-based therapies.

### Why It Is Important to do This Review

Research to date evaluating the efficacy of group-based interventions to treat PTSD has presented mixed evidence. Some research demonstrates that group-based interventions effectively lower PTSD symptoms in comparison to control conditions (e.g., waitlist control, treatment-as-usual; Barrera et al., 2013; Schwartze et al., 2019; Sloan et al., 2013). However, compared to individual-level treatments, group-based interventions have been shown to either be less effective (e.g., Ehring et al., 2014) or have similar effects on PTSD symptoms (e.g., Bisson et al., 2013; Schwartze et al., 2019; Sloan et al., 2013). Indeed, as previously highlighted, current policies and guidelines differ in the degree to which they recommend group-based treatment formats (e.g., International Society for Traumatic Stress Studies (ISTSS), 2018; Phoenix Australia Centre for Posttraumatic Mental Health, 2020). A consideration of the role of shared group membership and social identity processes that groups can harness may elucidate the efficacy of group-based interventions for treating PTSD. It may be the case that the effectiveness of group-based interventions depends on a number of considerations, specifically: (i) group-based factors (e.g., reported social support from group members, identification with other group members, facilitator characteristics), (ii) whether all group members have experienced a similar trauma (or a range of different traumas), (iii) if the trauma has stigma or shame attached, (iv) if the reason for the trauma was the result of intentional human action, and (v) the gender-composition of the group (mixed, women-only, men-only) in traumas with a clear gender dimension (e.g., sexual assault).

First, an up-to-date synthesis is needed comparing the efficacy of group-based psychological interventions to waitlist/usual care conditions, and to comparable individual-level treatment (i.e., the same psychological treatment approach is used), that includes both RCTs and non-RCTs.

Second, a number of group processes and factors are argued to bring about positive changes in well-being. For instance, a meta-analysis found that individuals who reported greater social identification with their therapy group demonstrated greater well-being (Steffens et al., 2021). Likewise, research on group treatments targeting anxiety, depression, and eating disorder symptoms have shown that individuals who reported greater identification with their group showed better outcomes (Cruwys, Alexander Haslam, et al., 2014; McNamara & Parsons, 2016). Borek et al. (2019) identified a number of group-based factors associated with success in health-behaviour-change interventions which may have important implications for group-based PTSD treatments, including group cohesion, group climate, group engagement, social support, and facilitator characteristics (e.g., warmth, relatedness, demographics). These studies highlight a number of important considerations that influence outcomes. Therefore, in this review we will describe any group-based and social identity factors reported on within each study and how they relate to PTSD. This may include, but is not limited to, perceived social support from other group members, measures of identification with other group members, likability of the group facilitator, and similarity between the group participants and the facilitator.

Third, the *fit* between the person and the other group members is an important factor in driving identification with the group (Cruwys et al., 2020), and thus the ability to benefit from the psychological and material resources groups offer (Jetten et al., 2012). In groups where individuals have all experienced a similar trauma this fit may (or may not) be better. Further, when all members of a group have shared a similar experience (‘a common fate’) this may foster greater social identification through a range of mechanisms including shared experience, shared values, solidarity, self-categorization, shared fate, and a sense of ‘in-group’ membership (Acharya & Muldoon, 2017; Drury, 2012; Muldoon, Haslam, et al., 2019). It may be the case that the effects of group treatments for PTSD vary between groups that are comprised of people who have all experienced the same trauma, and groups where individuals have experienced a range of different traumas. A consideration of the group composition in this way may be revealing in terms of treatment outcomes and therefore will be explored in this review.

Fourth, research has shown that experiencing a trauma with stigma attached to it can undermine psychological well-being further. Importantly, while diversity exists in cultural definitions of stigma, there is also remarkable consistency. Across many cultures and countries, certain experiences are universally associated with stigma. These include issues pertaining to sex and sexuality, infectious diseases/illnesses, and unfamiliar people and practices. A large body of research outlines the processes through which stigma can cause harm to health. Corrigan et al. (2011) proposed the most widely accepted model explaining the damage caused by stigma—the progressive model of self-stigma. This model distinguishes between enacted stigma and felt stigma. It is the latter that has the most powerfully negative consequences. Those affected by a stigmatized trauma are often aware of the stereotypes relating to their trauma ambient in their culture and then may also agree with it, apply it to themselves, and suffer harm as a result. In many regards then, this conceptualization of stigma is strongly related to stereotype endorsement of affected groups themselves—they themselves see their stigma as it were. For the purposes of this review, we conceptualize stigmatizing traumas in this regard, encompassing traumas such as rape, intimate partner violence, bereavement by suicide, and childhood sexual abuse. This is in comparison to other traumas which are less commonly associated with stigma (e.g., physical assault that is not domestic violence, witnessing an assault, bereavement due to accidents or illness, motor vehicle accidents, falls, natural disasters).

An examination of the efficacy of group-based treatments for stigmatizing traumas (vs. less stigmatizing traumas) is warranted considering conflicting evidence on group membership and stigma. On one hand, if all members of a group have experienced the same stigmatized trauma this can actually motivate people to engage with the group, which can have subsequent positive effects on well-being (Bradshaw & Muldoon, 2020; Lichtenstein, 2003). However, other research has shown that sometimes individuals may negatively self-stigmatize themselves or actively resist identifying with a stigmatized group, undermining any sense of belonging or connection with the group (Jetten et al., 2013; Muldoon, Haslam, et al., 2019; Quinn & Earnshaw, 2013). This then can negatively impact on well-being and increase distress, an effect termed the ‘social curse’ (Kellezi & Reicher, 2012). In this review, where groups are comprised of members who have all suffered a similar trauma, we will explore if stigma plays a role in treatment outcomes.

Fifth, past research demonstrates that traumas originating from intentional human acts, such as rape, combat exposure, childhood neglect, childhood physical abuse, sexual molestation, and physical assault, are associated with the highest rates of PTSD (Alisic et al., 2014; American Psychiatric Association, 2013; Charuvastra & Cloitre, 2008; Cloitre et al., 2019; Frans et al., 2005; Kessler, 1995). It is thought that these traumas in particular can compromise a person’s ability to engage and bond with others and undermine their sense of trust in others (Charuvastra & Cloitre, 2008). This may have implications for the effectiveness of group-based interventions in treating PTSD symptoms, as many group treatments involve participants sharing their thoughts and emotions with others in the group setting which typically requires some degree of trust. As such, research is needed to examine if trauma *type* (caused by intentional human actions vs. not caused by intentional human actions) moderates group treatment outcomes.

Sixth, the gender composition of treatment groups must be considered for traumas that have a gender dimension, for instance in the case of rape or domestic violence. From a social identity perspective, it may be beneficial for group members to be of the same sex so that members share potentially important and relevant social identities (S. A. Haslam et al., 2012; Muldoon, Haslam, et al., 2019). Indeed, two previous meta-analyses have indicated that the gender composition of the group is an important consideration. The results demonstrated that group-based interventions were more effective in alleviating PTSD symptoms in women-only groups (Schwartze et al., 2019; Sloan et al., 2013). Notably, in most of these studies (with women-only groups), the trauma experienced was gendered in nature (e.g., involved unwanted sexual contact, physical assault, sexual assault, childhood sexual abuse). This finding warrants further exploration to examine whether the gender composition of the treatment group affects clinical outcomes when the trauma is gendered.

Seventh, and finally, past research has suggested that the optimal group size for group treatments is six to eight people (Yalom, 1995). However, little research tests this recommendation. In the present meta-analysis, we will examine group size as a potential moderator.

The results of this systematic review and meta-analysis will inform policy and practice in the treatment of PTSD by providing insights into which conditions within group-based therapies for PTSD may be beneficial or detrimental for treatment outcomes. We build on the work of other recent systematic reviews which focused on group-based psychotherapeutic formal change theory interventions led by trained group leaders (Schwartze et al., 2019) and RCT treatments for PTSD (Lewis et al., 2020). We augment this prior work by including: any studies with a group-based intervention for PTSD regardless of the therapeutic approach taken, studies that are clinician-led and peer-led, and studies which do not use an RCT-design. The proposed review will provide an up-to-date synthesis of existing evidence and extend previous research by examining a range of moderators that may affect the efficacy of group-based treatments, including the nature of the trauma (interpersonal, stigmatized) and the group fit (in terms of gender and shared vs. unshared trauma). We also explore what, if any, group-based and social identity factors are recorded and how they relate to PTSD outcomes. As this review includes comparisons between group-based treatments, individual treatments, and/or no treatment, we will explore if comparison type (individual vs. no treatment) has an effect. The results will facilitate policy makers in coming to informed decisions surrounding treatment recommendations.

## Objectives

The primary objective was to assess the effects of group-based treatments on PTSD symptomology in people diagnosed with PTSD (by a clinician or screening instrument) or referred because of ongoing symptoms to a PTSD treatment group by a medical professional (see Table 1A for inclusion criteria). The objectives targeted by this review were:

(1) To compare the efficacy of group-based interventions compared to waitlist/usual care and/or minimal attention conditions that take place on an individual basis (i.e., not group-based).
(2) To compare the efficacy of group-based interventions compared to comparable individual-based treatment.
(3) To assess what social identity or group-based factors (if any) are measured and whether they are associated with the outcomes of interest.
(4) Where possible, assess whether the efficacy of group-based interventions is moderated by: shared experience of similar trauma (whether all members of the group experience the same trauma or different traumas), stigma (if the trauma/s experienced have societal stigma attached), the nature of the trauma (if the trauma was caused by the intentional actions of another person/s or not), gender composition of the group (mixed gender grouping, women-only, men-only), comparison type (individual-based treatment or no treatment), and group size (<6, 6-8, >8).
(5) To assess if there are any negative implications/adverse outcomes related to group-based treatments (e.g., worsening of PTSD symptoms, occurrence or worsening of other psychiatric symptoms such as depression and suicidal ideation, and/or increased or new alcohol or substance use).

## Methods

### Criteria for Considering Studies for This Review

#### Types of Interventions

##### Group-Based Interventions

For the full list of criteria to determine eligibility see Table 1A. Group-based treatments included a variety of formats as pre-registered, including group CBT, group counselling, and other group treatment formats. Consistent with a review by Schwartze et al. (2019), group-based treatment protocols included a minimum of three individuals per group and met for at least five sessions to be included. All interventions assessed PTSD symptomology pre-intervention (before or at the start of the first session) and post-intervention (either at the last session or after a follow-up period) using a valid and reliable measure. Subsequent follow-up measurements were included in the review when available, but more than one post-intervention measurement was not required for consideration in the review. Interventions could be delivered by a clinician or could be peer-led. Participants were not receiving any other psychological treatments for PTSD (e.g., individual therapy outside of the study). However, studies that included participants taking medication for PTSD (or related distress) were eligible for inclusion. To be considered for sub-group analyses, the type of trauma experienced by participants must have been explicitly stated in the study (or provided by the researchers when contacted for this information). Studies without this information were included to examine the overall effects of treatment type on PTSD outcomes but were not subject to subgroup analyses by trauma.

Sometimes, in the delivery of group treatments, participants attended individual-based sessions with a clinician. As pre-registered, we noted how many individual sessions these participants received. We planned to conduct sensitivity analyses to see if this influenced any observed results; however, only two studies met this criterion. While we planned to examine, where available, if group processes (e.g., social connectedness, group cohesion) influenced treatment outcomes, it was not a requirement for a study to report on these indices.

##### Control or Comparison Condition

To ensure meaningful comparisons, the goal was to examine the effectiveness of group-based interventions relative to comparison conditions which included either (i) an individual version of the same treatment, or (ii) a control condition where no active treatment was provided. In this regard, comparison individual-based treatments could include, but were not limited to, individual CBT, cognitive processing therapy, eye movement sensitization, stress inoculation training, individual counselling, psychodynamic therapy, and other individual treatments provided that this type of psychological treatment was comparable to the treatment employed in the group. In other words, both the individual and group conditions needed to employ a similar treatment (i.e., both used CBT, both used psychodynamic therapy). Where dissimilar treatments were used in the group condition and individual treatment condition (e.g., group CBT vs. individual psychodynamic therapy) these conditions were not compared in the meta-analysis. Control conditions could include, but were not limited to, waitlist groups, treatment-as-usual/usual care/standard care, symptom monitoring, and minimal attention control groups, where the control condition took place on an individual-level basis (i.e., not a group-based format). We had planned to include any study with a control condition delivered in a group format and to narratively describe the difference between this and the group treatment (i.e., group treatment vs. individual treatment, group treatment vs. individual control); however, this situation did not arise. Group-based control conditions are not comparable to individual-based control conditions as we believe groups themselves offer benefits. We further planned to include studies with two or more group-based interventions in the analyses if an appropriate comparison group (as specified) was also included within the study design; however, this situation did not occur.

#### Types of Studies

The focus of this review was on quantitative studies only; therefore, qualitative studies were not included. Participants must have been assigned to either a group-based treatment or a comparison condition. The comparison condition could have taken a number of forms including an active comparable individual-based psychological treatment and/or a control condition. Due to the criteria outlined in this review, we expected there to be a limited number of eligible RCTs, therefore both RCTs and non-RCTs were deemed eligible for inclusion. Examples of non-RCTs eligible for this review included: (i) quasi-RCTs where a quasi-random method of allocation was employed (e.g., order of recruitment) (ii) studies with a matching design to establish condition equivalence, or (iii) studies where randomisation was not employed, but baseline equivalence was ensured (e.g., via matching, statistical controls, or equivalence on PTSD symptoms), if not, the study needed to provide results from which baseline-adjusted effect sizes were calculated otherwise the study was excluded due to a critical risk of bias (see Section 4.3.6 for our definition of critical confounders). Our analyses were therefore conducted on all studies included, but sensitivity analyses were also conducted including only RCTS. Relevant studies available in English were included. Protocols, trial registrations, systematic reviews and meta-analyses, book chapters, letters to the editor, and conference proceeding were excluded. Due to the criteria outlined in this review, we expected there to be a limited number of eligible RCTs, therefore both RCTs and non-RCTs were deemed eligible for inclusion. Examples of non-RCTs eligible for this review included, quasi-RCTs, studies with a matching design, and studies where randomisation was not employed, but no baseline differences were observed between treatment and control conditions on key variables of interest (e.g., PTSD symptoms). Relevant studies available in English were included.

#### Type of Participants

All participants had to be over the age of 18 at the time of treatment to be included. At least 70% of participants in the sample had to have a diagnosis of PTSD, or screen positively for PTSD based on a psychometrically valid measure of PTSD (e.g. CAPS-5) or have been referred for treatment by a medical professional for their PTSD symptoms (similar to; Bisson et al., 2013; Lewis et al., 2020; Schwartze et al., 2019). No other restrictions in terms of age, location, ethnicity, severity of symptoms, duration of symptoms, length of time since trauma, or geographical location was applied. Samples where participants had comorbid disorders were eligible for inclusion, provided that the intervention was primarily aimed towards reducing PTSD symptoms (similar to; Simon et al., 2021).

#### Types of Outcome Measures

##### Primary Outcomes

The primary outcome measure was PTSD symptomology, or indices of post-traumatic stress symptoms (or clinical/base change), either indexed by self-report symptoms using a psychometrically valid PTSD scale or diagnosed by a qualified clinician (or both). To calculate changes in PTSD symptoms from pre-intervention to post-intervention, PTSD symptoms needed to be assessed using a continuous measure. Examples of self-reported scales we expected to include were the PTSD Checklist (Blanchard et al., 1996), the International Trauma Questionnaire (ITQ; Cloitre et al., 2018) and the Impact of Events Scale-Revised (IES-R; Weiss & Marmar, 1997). Clinician administered measures took precedence over self-reported measures, provided that the data was continuous in nature. Clinician rated measures, such as the Clinician Administered PTSD Scale for DSM-5 (CAPS-5; Weathers et al., 2018) is considered the gold standard for measuring PTSD symptoms, and preference for this type of measurement is consistent with past reviews (e.g., Bisson et al., 2013; Lewis et al., 2020). Primary outcomes were narratively described in the full review, with additional quantitative meta-analyses performed alongside subgroup analyses where appropriate.

##### Secondary Outcomes

Secondary outcomes were not required for inclusion in the review, but data was extracted on secondary outcomes of interest when reported. These included, where relevant, continuous measures of depression, somatic symptoms, and posttraumatic growth (or clinical/base change in these outcomes), as well as any adverse outcomes reported. We also reported descriptively on attrition (drop-out rates) and loss of PTSD diagnosis (i.e., remission). Validated peer-reviewed measures of these outcomes were eligible. For instance, depressive symptoms are often assessed via the Beck Depression Inventory (A. T. Beck, 1961; A. T. Beck et al., 1996), the Patient Health Questionnaire (Kroenke et al., 2001), the Centre for Epidemiologic Studies Depression Scale (Radloff, 1977), the Hamilton Depression Rating Scale (Hamilton, 1960), and the depression subscale of the Hospital and Anxiety Depression Scale (Zigmond & Snaith, 1983). Posttraumatic growth is often assessed using the posttraumatic growth inventory (Cann et al., 2010; Tedeschi & Calhoun, 1996). These outcomes were extracted for descriptive purposes and discussed narratively.

Where a sufficient number of studies reported on depression and posttraumatic growth as outcomes we had planned to run meta-analyses on these outcomes. We had also considered attrition as an outcome for subgroup analyses should a sufficient number of studies exist to allow for that to be examined. If so, we then planned to estimate how depression, attrition, and posttraumatic growth were impacted by the group-based factors previously outlined, specifically: trauma group type (same vs. mixed), stigmatized trauma (or not), if the trauma was caused by intentional human action/s (or not), gender grouping (mixed, women-only, men-only), and group size. We had also planned to explore, where possible, how reported group-based processes such as belonging, group cohesion, and trust, influence the primary outcome (PTSD symptoms) and if applicable, to discuss this narratively. However, none of these planned analyses were able to be conducted due to insufficient data.

#### Duration of Follow-Up

All studies reported on the primary outcome (PTSD symptoms) post-intervention. This follow-up was on the last day of treatment or soon afterwards (within a week/7 days). Where relevant, subsequent follow-up periods planned to be examined and discussed narratively with the aim of providing information on the temporal effects of group interventions on PTSD symptoms. Few studies reported such analyses, and these were therefore limited in the review (see Table 2).

#### Types of Settings

Group-based interventions that took place across a variety of settings were included. Examples of settings that group-based interventions took place in were community-based settings, hospital settings, in-patient treatment settings, out-patient treatment settings, residential treatment settings, prison settings, and clinical or medical settings. No restrictions were applied on country or locality (e.g., rural or urban).

### Search Methods for Identification of Studies

To identify studies, electronic databases were searched, and the reference lists of all included studies were screened for further potentially relevant research. For electronic searches, filters and limiters such as ‘English’, and ‘adults 18+’ were used where possible, for instance in Ovid Medline this was ‘all adult (19 plus years)’. Controlled vocabulary was used for variant spellings, truncations and wildcards. Search strategies were translated accordingly across the respective databases. Search terms were identified in consultation with our faculty librarian based on the PICOS framework. Tables 2A – 8A in the Supplementary Materials displays the search strategy tailored for each database. As an example, the search strategy tailored for Ovid Medline is presented in Table 1 below and is based on primary outcome AND intervention type AND study design. Terms for PTSD (line 2) and terms to locate studies employing a RCT design (line 5-8) were taken from a published Cochrane search strategy by Simon et al. (2021). To maximize the identification of studies not employing a RCT design, we utilized part of a search strategy published to identify non-RCTs (Waffenschmidt et al., 2020; lines 10–11), as well as terms used by Simon et al. (2021) to identify waitlist and/or treatment as usual designs (see line 9).

**Table 1.** Sample Search Strategy for Ovid MEDLINE Searches

1	exp stress disorders, post-traumatic/
2	(PTSD or ((posttrauma* or post-trauma* or post trauma*) adj3 (stress* or disorder* or psych* or symptom? or Neuroses)) or acute stress disorder* or combat disorder* or war neuros*).ti,ab,kf.
3	exp Stress disorders, post-traumatic/
4	(group* adj5 (treat* or intervention* or therap* or train* or program* or session* or setting* or factor* or process* or counsel* or psychotherap* or psychoeducat* or psycho-educat*)).mp.
5	Controlled clinical trial.pt.
6	Randomi#ed controlled trial.pt.
7	(RCT or at random or (random* adj3 (assign* or allocat* or control* or crossover or cross-over or design* or divide* or division or number))).ti,ab,kf.
8	trial.ab,ti,kf.
9	(control* and (trial or study or group*) and (placebo or waitlist* or wait* list* or ((treatment or care) adj2 usual))).ti,ab,kf,hw.
10	exp cohort studies/or exp epidemiologic studies/or exp clinical trial/or exp evaluation studies as topic/or exp statistics as topic/
11	((control and (group* or study or trial)) or (time and factors) or program or survey* or ci or cohort or comparative stud* or evaluation studies or follow-up*).mp.
12	or/1-2
13	or/3-4
14	or/5-11
15	12 and 13 and 14
16	Animals/not humans/
17	15 not 16
18	Limit 17 to (English language and “all adult (19 plus years)”)

#### Electronic Searches

Searches were conducted on electronic databases in April 2023 and re-run in May 2025. The following databases were searched: Embase (via the Elsevier platform), PsycINFO (via EBSCO), Cochrane Central Register of Controlled Trials (CENTRAL), MEDLINE (via Ovid), Applied Social Sciences Index and Abstracts (ASSIA), PTSDPubs (via ProQuest for dissertations, formerly PILOTS) and ProQuest Dissertations. Speciality journals were hand searched, including the European Journal of Psychotraumatology, the Journal of Traumatic Stress, and Psychological Trauma: Theory, Research, Practice and Policy. We also searched preprint servers (PsyArXiv and medRxiv) for unpublished literature. We planned to search for other grey literature on Open Access Theses and Dissertations, however the server is no longer functioning. Electronic searches were also conducted across several recommended websites (Kugley et al., 2017), to further identify any sources of grey literature, including: the Government of Canada publications (https://publications.gc.ca/), Grey Literature Report (https://www.greylit.org/), and the National Institute for Health and Care Excellence (NICE; https://www.nice.org.uk/), as well as the PTSD Trials Standardized Data Repository (PTSD-Repository; https://ptsd-va.data.socrata.com/).

Searches of relevant systematic reviews and meta-analyses reference lists were also conducted (e.g. Schwartz et al., 2019; Sloan et al., 2013). We also searched reference lists of included papers to identify any additional studies that may be eligible for inclusion. To search for unpublished articles that may not have been listed on the specified databases we put a call out on Twitter (now X; mentioning organizations that focus on PTSD), on our research group accounts and our own personal accounts, as well as our laboratory website (https://growth-ul.wixsite.com/psychology), asking for information on unpublished studies or data that aligned with the aims of our review. We contacted international researchers by posting a call for information on the electronic mailing lists of a number of professional psychology societies and asked these societies to share this call with their members, such as the Society of Personality and Social Psychology, the American Psychosomatic Society, the Society of Affective Science, the European Association of Social Psychology, the Stress, Trauma, Anxiety, the International Society for Traumatic Stress Studies, and the European Society of Traumatic Stress Studies.

### Data Collection and Analyses

#### Description of Methods Used in Primary Research

We anticipated that most studies would employ an RCT of some form that would consist of a group-based treatment intervention condition and some other type of comparison condition (e.g., an individual-based treatment or a control/waitlist/usual care condition). However, we also expected quasi-experimental designs to be employed due to the logistics of RCTs. For instance, participants may have been allocated to a group-based intervention or a waitlist based on recruitment timing (e.g., participants allocated to the intervention, then assigned to the waitlist once the intervention group is full). Studies needed to assess PTSD symptoms pre- and post-intervention.

#### Criteria for Determination of Independent Findings

Potential issues were previously identified and planned for in this review. First, where multiple publications existed using the same data, only the most complete report of this data was included. Second, we used a correlated-hierarchical effects (CHE-RVE) model (Pustejovsky & Tipton, 2022) to account for dependencies in mean effects in cases where studies included multiple outcome measures assessing the same construct or included multiple intervention arms (e.g., two group treatments and one control). For studies that included multiple intervention arms, we included the intervention arms/conditions that met the eligibility criteria. To account for the non-independence of effect sizes within studies, we applied robust variance estimation with the clubSandwich package in *R*, specifying clustering at the study level. A constant sampling correlation (ρ = 0.6) was assumed to model the correlation between effect sizes within clusters, as recommended for meta-analyses with dependent effect sizes (Fisher & Tipton, 2015).

#### Selection of Studies

**Figure 1.**
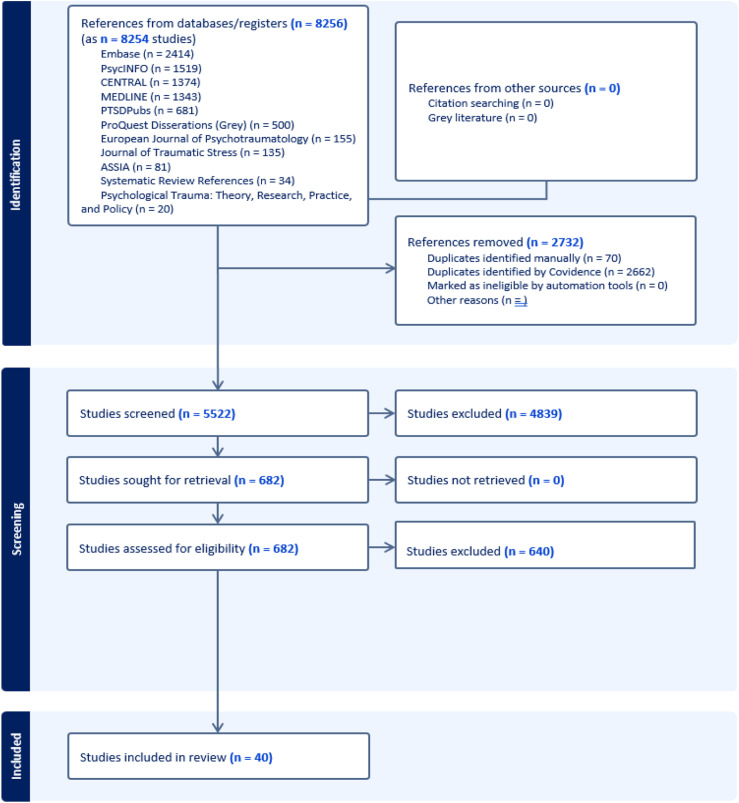
PRISMA flow diagram

Once searches were completed, all retrieved studies were stored on Covidence software and duplicate studies were removed. Following deduplication, the title and abstract of all studies were independently screened by two reviewers and compared against eligibility criteria. At this point, studies that clearly did not meet the inclusion criteria were excluded (e.g. not a group-based intervention, group-based intervention for children). If a study was deemed eligible by one author (but not both), or if a study’s eligibility was unclear from the title and abstract, then these studies were rescreened in the full text screening phase. Studies that were deemed eligible underwent full text screening for eligibility, again screening was conducted independently by two reviewers. In case of disagreements, these were resolved through discussion and consensus, and when necessary, by asking a third author to screen the study independently to reach a majority decision. By completing the preliminary screening and full text screening independently this helped minimize bias. Both screening processes were piloted by the review team beforehand. The full screening process, including reasons for exclusion, is documented and presented in Figure 1 (PRISMA, 2020 flow chart; Page et al., 2021).

#### Data Extraction and Management

Data extraction was carried out by two independent reviewers to reduce bias and potential errors. A template was developed, informed by the data extraction tool on Covidence software, and additional data was added based on the criteria for this review and the variables of interest. This data extraction sheet was piloted with a random sample of 10 records to assess its validity. The following data was extracted: (i) study metadata (authors, title, year, journal of publication), (ii) study characteristics (study design, sample size, study setting, participants per group, average group size), (iii) participant information (age, gender, and any other relevant information), (iv) trauma characteristics (all participants experienced the same trauma or a mix of traumas, if the trauma was caused by intentional human action/s or not, if the trauma could be perceived as stigmatized or not), (v) intervention information (type of treatment used, assignment to intervention, number of participants per group, number of group sessions, duration of each group session, type of facilitator (clinician or peer), number of facilitators (vi) gender of participants in the group intervention and comparison condition (mixed, women-only, men-only), (vii) comparison condition information (type of control, duration), (viii) primary and secondary outcomes (effect sizes, outcome measures, type of measure), (ix) any group-based factors (e.g., social support, group cohesion, trust), and limitations of the study where applicable.

#### Assessment of Risk of Bias

To assess bias within studies, we used the Revised Cochrane risk-of-bias tool for randomized trials (RoB 2; Sterne et al., 2019) for RCTs and the Risk of Bias in Non-randomized Studies—of Interventions (ROBINS-I; Sterne et al., 2016) for non-RCTS. Two reviewers independently assessed studies for risk of bias and any disagreements were assessed by a third reviewer. For the RoB 2, an overall risk of bias rating of low risk, some concerns or high risk was given to studies based on criteria from five different domains, if a study received a rating of ‘high risk’ in one domain, it received a rating of **‘**overall high risk of bias’. For the ROBINS-I, an overall risk of bias rating of low, moderate, serious or critical risk was given to studies based on criteria from seven different domains, with the overall risk of bias rating determined by the highest level of risk in any domain. Studies judged as having a critical risk of bias using the ROBINS-I would be excluded from the meta-analysis, consistent with recommendations (Sterne et al., 2016). As there is no critical level of risk within the RoB 2 tool any RCTs judged as being too problematic to provide useful evidence of the intervention would be excluded from the meta-analysis, similar to past reviews (Dalgaard et al., 2022). Consistent with this approach (Dalgaard et al., 2022), if a serious risk of bias was determined in multiple domains within the RoB 2 or ROBINS-I tool then this may lead to the study being deemed as overall critical risk of bias and excluded from the meta-analysis. However, no studies were deemed to be at critical risk of bias.

As we were interested in the group nature of the treatments, methodological heterogeneity existed across studies in terms of the type of psychological treatment employed, the length of follow-up, and the way in which groups were facilitated, therefore we planned for studies with an overall high risk of bias to be included. However, to see if it affected results, we planned to conduct sensitivity analyses by excluding studies at a higher overall risk of bias. For the purposes of this review, higher overall risk of bias is indicated if the study is deemed ‘overall high risk of bias’ using the RoB 2 tools or ‘overall serious risk of bias’ using the ROBINS-I tool (see Sensitivity Analyses plan).

#### Confounding

We identified PTSD symptomology at baseline as a critical confounder. For RCTs we planned for this in our analyses (e.g., preference to baseline adjusted effect sizes, using the pre–post-test correlation when adjusted effect sizes are not reported). For non-RCTs, these studies needed to ensure baseline equivalence, otherwise the study needed to provide results from which baseline-adjusted effect sizes were calculated; if not, the study would be excluded due to an overall critical risk of bias (ROBINS-I). Baseline equivalence was assessed using the statistical comparisons reported in the original studies (e.g., t-tests, ANOVAs). No studies were excluded for baseline imbalance.

#### Measures of Treatment Effect

Only studies that reported continuous measures of PTSD were eligible for inclusion. Both within-group effect sizes and between-group effect sizes, and 95% confidence intervals, were calculated. Between-group effect sizes were calculated for each comparison and the primary outcome of interest (PTSD symptoms). Effect estimates were quantified as the standardized mean difference (SMD) by extracting the relevant data (means, sample sizes, standard deviations). Between-group effect sizes were computed using Hedges adjusted g using the small sample size bias correction (Hedges & Olkin, 1985), see equation (1), where the difference between the mean outcome for the intervention and the comparison group is divided by the pooled within-group standard deviation. Use of the standardized mean difference allows for comparisons to be made across groups when variables are not operationalized in the same way.

$$SMD=\frac{M_{G1}-M_{G2}}{Sp}\;\left(1-\frac{3}{4N-9}\right)$$

Where possible, we used alternative available data to calculate effect sizes when means and *SD*s were not reported, using the methods and tools suggested by Lipsey and Wilson (2001) and Pustejovsky (2016). However, in some cases, studies did not provide sufficient data and therefore it was not possible to calculate effect sizes for these studies.

To be considered clinically relevant, the group-based intervention needed to demonstrate an effect size of >0.80 for waitlist and usual care comparisons, >0.5 for attention control comparisons, >0.4 for placebo control comparisons, and >0.2 for active treatment comparisons, consistent with the definition of clinical importance (International Society for Traumatic Stress Studies [ISTSS], 2018; Lewis et al., 2020). Comparator type was modelled as a categorical moderator (g ∼ 0 + Ctype), allowing separate pooled effect size estimates for each control group category. Clinical importance was then evaluated relative to the ISTSS benchmark corresponding to each comparator type.

In other cases, multiple effect sizes were extracted from studies that employed more than one measure of PTSD symptoms. Therefore, to account for dependency in the data we used a Correlated and Hierarchical Effects Model (CHE-RVE; Pustejovsky & Tipton, 2022), in line with the decision tree presented in Pustejovsky and Tipton (2022). We used Microsoft Excel to store data, and *R* Statistical Software (R Core Team, 2021) to conduct statistical analysis.

#### Unit of Analysis Issues

Effect sizes were computed for outcomes within each study. Where a study provided more than one effect size for each outcome, we accounted for this in statistical analyses. If there were more than two intervention arms, we planned to only include those that met the review criteria. Participants may have been randomized, or allocated, into the group-based intervention or comparator groups in clusters. To minimize bias, we applied a cluster bias correction (WWC, 2021), in addition to the small sample size adjustment.

#### Dealing With Missing Data

Study authors were contacted in cases where data of interest were not reported. Where data was not available (e.g. means and standard deviations), we did not impute values. In the case of missing data due to follow-up attrition (e.g., from pre-to post-intervention) we followed the principles of intention-to-treat analyses as much as possible. We report on the extent of missing data narratively in the review and in relation to the risk of bias.

#### Subgroup Analysis and Investigation of Heterogeneity

We examined if the following factors affected the observed results and explained any observed heterogeneity: (i) trauma-group type (members of the group experience the same trauma vs. different traumas or multiple traumas), (ii) stigma (the trauma experienced is stigmatized vs. not), (iii) the nature of the trauma (caused by the intentional actions of another person/s vs. not), (iv) gender grouping of the group-based treatment (mixed gender grouping vs. women-only vs. men-only) and (vi) group size (<6, 6-8, >8). Where study characteristics spanned more than one predefined subgroup category (e.g., group size ranges), studies were assigned to a single category based on the reported mean value. We also ran subgroup analysis to explain observed heterogeneity between studies based on the type of treatment (e.g. GCBT or GCPT) and the type of comparator (e.g., control or active control) used within studies. Due to variation in the type of treatment used between studies, and for the purpose of analysis, we collapsed treatment type into the following six subgroups: group cognitive behavioural therapy (*n* = 7), group cognitive processing therapy (*n* = 6), movement-based treatments (*n* = 6), group psychotherapy (*n* = 5; interpersonal/psychotherapeutic group therapies not otherwise specified, e.g., group counselling, interpersonal psychotherapy), alternative treatments (e.g. MBSR, music therapy; *n* = 7) and other group therapy, in cases where the treatment type was used in ≤3 studies (e.g. GACT, *n* = 3; DBT, *n* = 1). We had planned to run subgroup analysis to examine if the group was led by a clinician/facilitator or a peer contributed to heterogeneity, however we were unable to do so as all of the groups were facilitated by a clinician/facilitator and none of the treatments were facilitated by peers. We also planned to run subgroup analysis to assess the role of gender where traumas had a clear gender dimension (e.g. sexual assault), however, all of these groups were women only, so it was not possible to run this analysis. Instead, we assessed the role of gender composition across all groups.

We assessed the presence and extent of between-study heterogeneity in a number of ways. In terms of methodological heterogeneity, this was discussed narratively within the review as a range of group-based treatments were used across studies. We anticipated some degree of heterogeneity due to methodological differences across group-based intervention treatments. Therefore, for our analyses we used CHE(-RVE) models which accounts for both within-study heterogeneity (ω) and between-study heterogeneity (τ), and we report on this data. A forest plot aligned with dependent effect sizes is used to visualize the extent of heterogeneity (by examining the width of confidence intervals and degree of overlap), in line with Winters et al. (2022).

#### Sensitivity Analysis

Before conducting our analysis, we checked for outliers in the distribution of effect sizes. In line with our protocol, outliers were defined as effect sizes falling more than three times the interquartile range (IQR) below the first quartile (Q1 − 3×IQR) or above the third quartile (Q3 + 3×IQR) (Tukey, 1977; Winters et al., 2022). Kara et al. (2025) exceeded the upper bound of this criterion and therefore met our predefined definition of an outlier. Consistent with the protocol, we re-ran the meta-analysis with and without this study to assess its impact on heterogeneity. Removal of Kara et al. (2025) reduced heterogeneity from *I*^*2*^ = 88.7% (Q = 327.08, df = 37) to *I*^*2*^ = 87.6% (Q = 289.45, df = 36) and was therefore removed from our analysis. We also planned to remove studies deemed at overall high risk of bias based on RoB 2 ratings or overall serious risk of bias based on ROBINS-I ratings (see Assessment of risk of bias).

#### Treatment of Qualitative Research

This review did not include qualitative research as this was not the focus of the present review.

#### Reporting Bias

Egger’s test for funnel plot asymmetry was used to test for possible publication bias, see Figure 6 (Egger et al., 1997). Risk of bias was assessed using ROB 2 and ROBINS-I, as previously described.

## Results

### Description of Studies

#### Results of the Search

**Figure 2.**
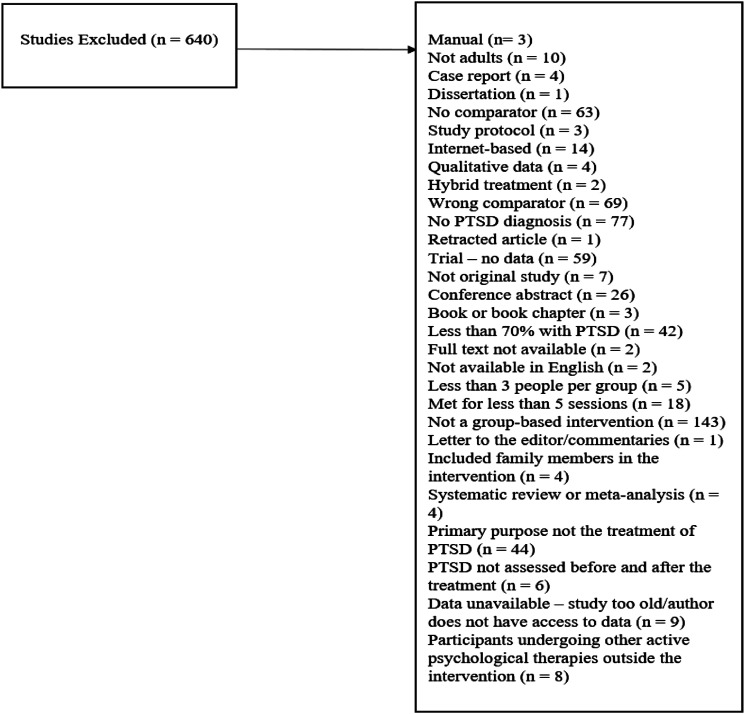
Reasons for excluding studies

Our search identified 8,256 results (see Figure 1 for PRISMA flow diagram). During de-duplication, 2,662 studies were removed. Following title and abstract screening, 4,839 studies did not meet eligibility criteria (see Figure 2 for exclusion reasons). The full texts of 682 studies were screened and of these, a total of 40 studies were deemed eligible and included in this review.

#### Included Studies

Study characteristics are summarised in Tables 2 and 3. Of the included studies, the majority (*n* = 26) were conducted in the USA, three were conducted in Iran, two were conducted in the Netherlands, two were conducted in Turkey, and one was conducted in Australia, Austria, China, Jordan, South Africa, Sweden, and the UK, respectively. The weighted mean age of participants across studies was 43.3 years. Most studies were conducted with women-only (*n* = 16) or mixed (*n* = 16) groups, only five studies were conducted with men, and three studies did not report on the gender composition of their groups. The mean number of participants per group-based treatment was ten. The majority of interventions targeted people with combat-related PTSD (*n* = 15), followed by interventions for people with experiences of abuse, both physical and sexual (*n* = 6), people with a combination of different traumatic experiences (*n* = 6; studies where participants experienced a range of different traumas), people with multiple traumatic experiences (*n* = 5; studies where participants experienced repeated or numerous traumas), people who experienced motor vehicle accidents (*n* = 2), women with PTSD related to a cancer diagnosis (*n* = 1), people who experienced an earthquake (*n* = 1), people who experienced a car-hijacking (*n* = 1), refugees (*n* = 1), and mothers who experienced a birth-related trauma (*n* = 1). Most group-based treatments used either CBT, CPT, group psychotherapy, or alternative approaches. Comparators were either active controls (participants received the same treatment as the intervention but delivered in an individual rather than group format), controls (participants received no intervention or minimal attention) or waitlist controls (participants did not receive the intervention initially but were offered it after the study period), or treatment as usual conditions (participants received standard care).

**Table 2.** Characteristics of Included Studies

Authors	Setting	M. Age	Gender	Treatment	Control	Type of trauma	No. Sessions	No. per group	Secondary outcome^a^	Follow-up	Study design
Akbarian et al. (2015) (Iran)	Hospital	30.9	Mixed	GCBT	Control	Mixed	10	14	Depression	None	RCT
Ali (2020) (Jordan)	Refugees	​	Women	Group therapy	Control	Displaced	14	Not reported	None	None	Quasi-experimental
Beck et al., 2009 (US)	MVA, Memphis	43.3	Mixed	GCBT	Control	MVA	14	17	Depression	None	RCT
Bektaş-Aydın & Yüksel-Şahin (2025)	University, Turkey	22.8	Women	GACT	WC	IPV	12	10	None	1 month	Quasi-experimental
Bormann et al. (2012) (US)	VA Unit, San Diego	57.3	Mixed	Mantram	Control	Combat related	6	Not reported	None	None	RCT
Bormann et al. (2008) (US)	VA Unit, San Diego	56	Men	Mantram	WC	Combat related	6	14	None	None	RCT
Capone et al. (2018) (US)	VA Unit, Rhode Island	34.3	Mixed	GCBT	TAU	Combat related	1–4 individual 5–12 group	8	None	3 months	RCT
Carr et al. (2012) (UK)	Specialised NHS Centre	39	Mixed	Music therapy	WC	Mixed	10	9	Depression	None	RCT
Carter et al. (2013) (AUS)	​	58.4	Men	Yoga	WC	Combat related	22hrs/5 days	14	Depression	6 months	RCT
Castillo et al. (2016) (US)	VA Hospital, Texas	35.9	Women	GCBT	Control	Mixed	16	Not reported	None	3 and 6 months	RCT
Chard (2005) (US)	VA Medical Centre, Cincinnati	32.8	Women	GCPT	Control	CSA	17; individual and group	Not reported	Depression	3 months and 1 year	RCT
Classen et al. (2001) (US)	Hospital and Church, San Francisco	38.4	Women	Trauma and present focused therapy	WC	CSA	24	7	None	None	Pilot
Dorrepaal et al. (2012) (NL)	Mental Health Institute	38.5	Not reported	GCBT	TAU	CA	20	8–12	None	None	RCT
Drozdek et al. (2012) (NL)	Trauma treatment facility for PTSD	38.1	Men	Group therapy	WC	Combat related	85 sessions over 1 year	27; 22; 7	Depression	None	Observational
Falsetti et al. (2001); 2008 (US)	University and Rape Crisis Centre, South Carolina	35.1	Women	M-CET	WC	Multiple	12	7	Depression	None	Pilot
Goldstein et al. (2018) (US)	VA Medical Centre, San Francisco	46.8	Mixed	Exercise	WC	Not reported	36	16	None	None	RCT
Grove et al. (2021) (US)	VA Health Centre, Virginia	44.9	Mixed	REBT	TAU	Combat related	5	Not reported	Depressive symptoms	None	Evaluation
Heath (2009) (US)	Women’s Correctional Facility, Idaho	35	Women	Seeking Safety	WC	Mixed	22	11–18	None	None	Quasi-experimental
Hetz (1999) (SA)	​	​	Not reported	GCBT	WC	Car hijacking	12	Not reported	Depression	None	Quasi-experimental
Johnson (1990) (US)	Family Centre, California	33	Women	Group therapy	WC	SA	12	8	None	None	Quasi-experimental
Kara et al. (2025) (Turkey)	Support Centre, Istanbul	27.9	Mixed	Group therapy	Control	Earthquake	8	10	Depression	None	RCT
Kearney et al. (2013) (US)	VA health Centre, Seattle	52	Mixed	MBSR	TAU	Multiple	9	5–10	Depression	4 months	RCT
Kent et al. (2011) (US)	VA Health Centre, Arizona	54	Mixed	Resilience treatment	WC	Mixed	12	10	Depression	None	RCT
King et al. (2013) (US)	VA Health Centre, Michigan	59	Not reported	MBCT	TAU	Combat related	8	5	None	None	Feasibility
Koochaki et al. (2018) (Iran)	Hospital	23.4	Women	GCBT	TAU	NICU	8	Not reported	None	3 weeks	RCT
Krupnick et al. (2008) (US)	Low-income Centres, Washington	32	Women	Group therapy	WC	PA and SA	16	3–5	Depression	4 months	RCT
Mitchell et al. (2014) (US)	VA Medical Centre, Massachusetts	44.4	Women	Yoga	WC	Multiple	12	4–5	Depressive symptoms	1 month	RCT
Nagrath (2020) (US)	Research Institute, California	48.7	Mixed	EAT	TAU	Combat related	10	8–10	None	None	Quasi-experimental
Oppenauer et al. (2023) (Austria)	Hospital for psychosomatic Medicine	43	Mixed	DBT	TAU	Mixed	60	Not reported	None	None	Quasi-experimental
Ragsdale et al. (1996) (US)	Specialized PTSD program, Virginia	43.3	Mixed	Group therapy	WC	Combat related	26 day treatment	24	None	None	Quasi-experimental
Resick & Schnicke (1992) (US)	​	30.6	Women	GCPT	WC	SA	12	5-8	Depression	3 and 6 months	Quasi-experimental
Resick et al. (2017) (US)	Research Clinic, Texas	33.2	Mixed	GCPT	AC	Combat related	12	8–10	Depression	6 months	RCT
Rudstam et al. (2022) (Sweden)	Trauma Centre	43.7	Women	Music therapy	WC	PA and SA	12	5–7	Depression	None	RCT
Sarizadeh et al. (2021) (Iran)	Cancer Centre	48.2	Women	GACT	WC	Cancer diagnosis	8	Not reported	None	None	RCT
Seppälä et al. (2014) (US)	Not reported	28.7	Men	Yoga	WC	Combat related	7 days	11	None	1 month and 1 year	RCT
Spiller et al. (2023) (US)	Multiple VA residential centres across the US	44	Mixed	GCPT	AC	Multiple	Minimum 8	Varied	None	4 months	Observational
Wharton et al. (2019) (US)	VA PTSD Clinic, California	54.5	Men	GACT	AC	Combat related	12	10	None	None	Pilot
Williams et al. (2022) (US)	VA Medical Centre, Texas	33.9	Mixed	GCPT	WC	Combat related	12	4–5	Depression	None	Quasi-experimental
Yi et al. (2022) (China)	Mental Health Centre	41.2	Women	Yoga	Control	MVA	6	Not reported	Depression	3 months	RCT
Zlotnick et al. (2009) (US)	Residential SUD programme, Rhode Island	34.6	Women	Seeking Safety	TAU	Multiple	18–24	3–5	None	3 and 6 months	RCT

^a^Secondary outcomes refers to the secondary outcomes of interest in this review (i.e. depression, psychosomatic symptoms, post-traumatic growth), not all secondary outcomes included in each study.

**Table 3.** Summary of findings

Authors	Same trauma	Intentional	Clinician/Facilitator-led or peer-led	Social-identity/Group-based factors measured	Additional social/Group factors
Akbarian et al. (2015) (Iran)	No	Yes	Clinician/facilitator	None reported	None reported
Ali (2020) (Jordan)	Yes	Yes	Clinician/facilitator	None reported	The authors reported conducting three focus group meetings to explore the everyday challenges experienced by participants living as refugees with PTSD. The intervention emphasised group processes, including hope installation and socialisation techniques. Feelings of commonality and belonging experiences were also employed.
Beck et al., 2009 (US)	Yes	No	Clinician/facilitator	None reported	The authors reported that the treatment was guided by group therapy principles, including strategies designed to enhance group cohesion.
Bektaş-Aydın & Yüksel-Şahin (2025) (Turkey)	Yes	Yes	Clinician/facilitator	None reported	The techniques implemented in the program were specifically adapted to focus on women and IPV.
Bormann et al. (2012) (US)	Yes	Yes	Not reported	Measured participants’ identification with religion and/or spirituality	Measures included social factors
Bormann et al. (2008) (US)	Yes	Yes	Not reported	None reported	None reported
Capone et al. (2018) (US)	Yes	Yes	Clinician/facilitator	None reported	The authors stated that the treatment was adapted for veterans and informed by previous work.
Carr et al. (2012) (UK)	No	Yes	Clinician/facilitator	None reported	The authors encouraged verbal reflection on thoughts and feelings arising from the musical experience.
Carter et al. (2013) (AUS)	Yes	Yes	Clinician/facilitator	None reported	The authors modified the programme for a veteran population and highlighted the role of group processes in facilitating the sharing of personal life stories
Castillo et al. (2016) (US)	No	Yes	Not reported	None reported	None reported
Chard (2005) (US)	Yes	Yes	Clinician/facilitator	None reported	The authors reported that recreating social interactions allowed participants to process their thoughts and feelings with someone other than their therapist.
Classen et al. (2001) (US)	Yes	Yes	Not reported	None reported	The authors reported that the intervention emphasised establishing a safe and trusting group environment, encouraging members to share their experiences and engage in group processes that supported interpersonal learning.
Dorrepaal et al. (2012) (NL)	Yes	Yes	Clinician/facilitator	None reported	The authors reported that the group format aimed to promote hope and support participants in reframing trauma-related symptoms as understandable responses to trauma, with the goal of reducing shame, guilt, and isolation.
Drozdek et al. (2012) (NL)	Yes	Yes	​	None reported	The authors stated that a specific trauma-focused treatment model for asylum seekers and refugees was used, known as the Den Bosch treatment model.
Falsetti et al. (2001) (US)	No	Yes	Clinician/facilitator	None reported	None reported
Goldstein et al. (2018) (US)	No	Yes	Clinician/facilitator	None reported	None reported
Grove et al. (2021) (US)	Yes	Yes	Not reported	None reported	None reported
Heath (2009) (US)	No	Yes	Clinician/facilitator	Used the Seeking Safety Adherence scale to assess the group facilitators’ treatment adherence and group members’ sense of therapeutic alliance. Additionally, several items assessed overall group processes and cohesiveness. However, this measure has not been evaluated psychometrically, it was created specifically to address treatment adherence for *Seeking Safety*.	The authors reported that the programme addressed interpersonal processes within the group. The scale assessed whether facilitators created a safe and supportive environment, demonstrated care and compassion, and managed crises or conflict within the group.
Hetz (1999) (SA)	Yes	Yes	Not reported	None reported	Participants met informally before sessions without the therapists present. The authors reported that, for many participants, this represented their first opportunity to meet others with PTSD, and recognising shared experiences contributed to group cohesion.
Johnson (1990) (US)	Yes	Yes	Clinician/facilitator	None reported	None reported
Kara et al. (2025) (Turkey)	Yes	No	Clinician/facilitator	None reported	The authors reported that sessions were designed to promote rapport, strengthen connections between participants, and explore shared and differing experiences. Participants’ sharing of personal experiences was described as supporting trust and bonding, with group members reflecting on changes they experienced during the intervention in the final session.
Kearney et al. (2013) (US)	No	Yes	Clinician/facilitator	None reported	None reported
Kent et al. (2011) (US)	No	Yes	Clinician/facilitator	None reported	None reported
King et al. (2013) (US)	Yes	Yes	Clinician/facilitator	None reported	The authors adapted the MBCT treatment protocol for combat-related PTSD.
Koochaki et al. (2018) (Iran)	Yes	No	Clinician/facilitator	None reported	The authors stated that the focus of the group was on building relationships between mothers with shared experiences.
Krupnick et al. (2008) (US)	No	Yes	Clinician/facilitator	None reported	The authors stated that the initial phase aimed to establish group cohesion.
Mitchell et al. (2014) (US)	No	Yes	Clinician/facilitator	None reported	None reported
Nagrath (2020) (US)	Yes	Yes	Clinician/facilitator	None reported	The authors stated that the EAP included group reflection activities and culturally sensitive activities.
Oppenauer et al. (2023) (Austria)	No	Yes	Clinician/facilitator	None reported	None reported
Ragsdale et al. (1996) (US)	Yes	Yes	Not reported	None reported	The authors stated that the activities were centred around building trust and group problem solving to create a supportive environment.
Resick & Schnicke (1992) (US)	Yes	Yes	Clinician/facilitator	None reported	The authors stated that the focus of the group was on creating a trusting environment.
Resick et al. (2017) (US)	No	Yes	Clinician/facilitator	None reported	The authors tailored CPT to a veteran population.
Rudstam et al. (2022) (Sweden)	No	Yes	Not reported	None reported	The authors adapted the treatment to a trauma treatment with an emphasis on building safety and group cohesion.
Sarizadeh et al. (2021) (Iran)	Yes	No	Not reported	None reported	None reported
Seppälä et al. (2014) (US)	Yes	Yes	Clinician/facilitator	None reported	The authors state that treatment groups included periods of group discussion.
Spiller et al. (2023) (US)	No	Yes	Clinician/facilitator	None reported	None reported
Wharton et al. (2019) (US)	Yes	Yes	Clinician/facilitator	None reported	None reported
Williams et al. (2022) (US)	Yes	Yes	Clinician/facilitator	None reported	Authors noted that veterans with high levels of social isolation and low connectedness may particularly benefit from group-based interventions.
Yi et al. (2022) (China)	Yes	No	Clinician/facilitator	None reported	None reported
Zlotnick et al. (2009) (US)	No	Yes	Clinician/facilitator	None reported	None reported

#### Excluded Studies

A further 640 studies were excluded from the review following full text screening (see Figure 2). The most common reasons for excluding studies were because they were not group-based interventions (i.e. individual interventions), less than 70% of participants had a diagnosis of PTSD, used the wrong comparator (e.g. group CBT vs psychoeducation), or no comparator, or participants were engaging in another active treatment. For example, a study by Kananian et al. (2020) was excluded because only 58% of participants had a diagnosis of PTSD, similarly in a study by Levine et al. (2005) less than 70% of participants had a diagnosis of PTSD. Burton et al. (2019) evaluated the efficacy of equine assisted therapy for veterans with PTSD; however, this study was excluded because participants were engaging in another active treatment at the time of the intervention.

### Risk of Bias in Included Studies

**Figure 3.**
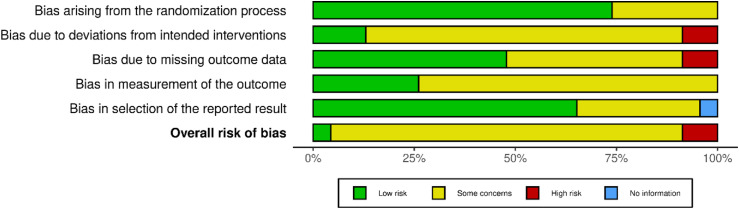
RoB-2 summary plot

**Figure 4.**
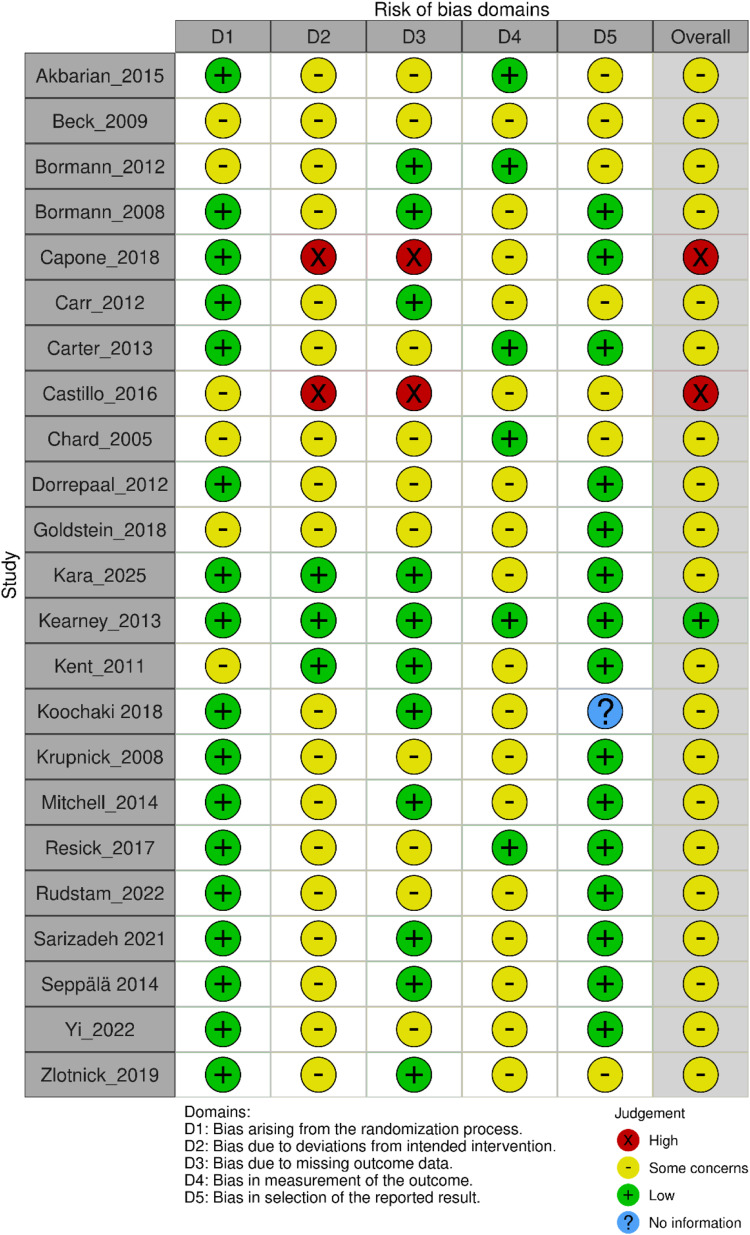
RoB-2 traffic light plot

To assess bias within studies, we used the RoB 2 (Sterne et al., 2019) for RCTs and the ROBINS-I (Sterne et al., 2016) for non-RCTS. Of the 40 included studies, 23 were RCTs and 17 were non-RCTs. Of the 23 RCTs assessed using the RoB 2, two studies were deemed at overall high risk of bias (Capone et al., 2018; Castillo et al., 2016). Both of these studies were rated high risk based on a probably yes rating in category D2 ‘deviations arose because of trial context; these deviations likely affected the outcome and were unbalanced between groups.’ For example, Capone et al. (2018) stated that *‘‘it is possible that our use of a combined individual/group format contributed to the dropout rate we observed’’,* and Castillo et al. (2016) stated that *‘‘n = 6 voluntarily dropped out of treatment due to stress and desiring other treatment.’’* One RCT study (Kearney et al., 2013) was rated as overall low risk of bias and the remaining 20 RCTs were rated as having some concerns. For RoB 2 ratings see Figures 3 and 4.

**Figure 5.**
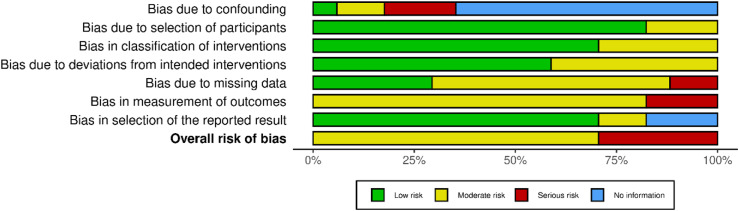
ROBINS-I summary plot

**Figure 6.**
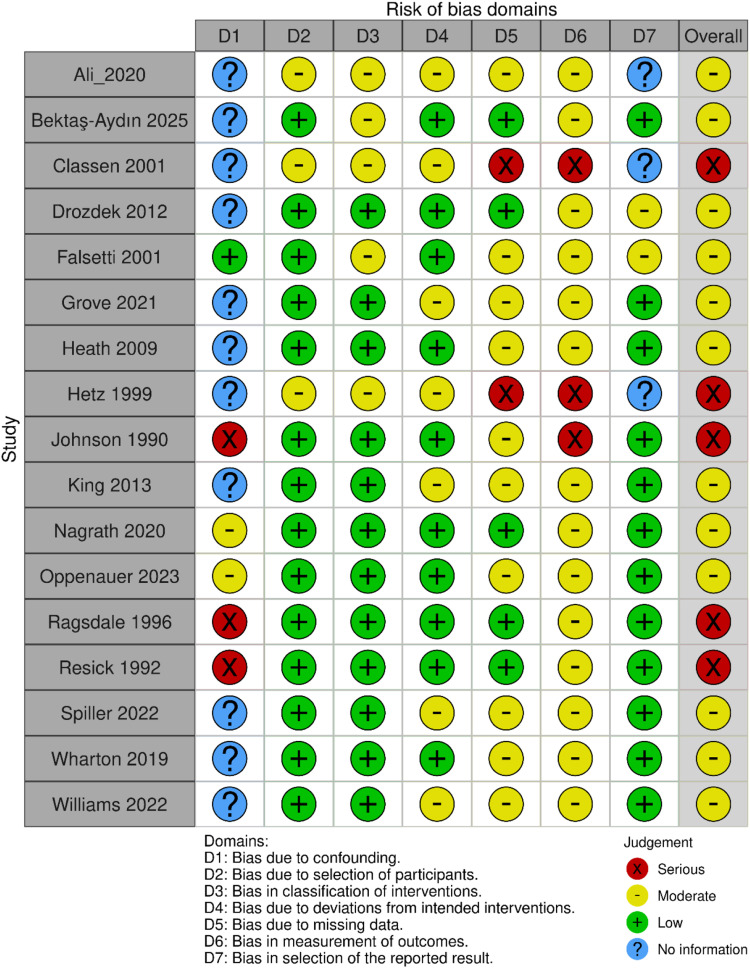
ROBINS-I Traffic Light Plot

Risk of bias for the 17 non-RCTs was assessed using the ROBINS-I. Twelve studies were rated as overall moderate risk of bias, and five studies were rated as overall serious risk of bias. Johnson (1990) received an overall rating of serious risk in category D6 because they developed their own scale to measure PTSD symptoms. Both Ragsdale et al. (1996) and Resick and Schnicke (1992) received a rating of serious/no information in category D1 due to potential confounding of the effect of the intervention. Hetz (1999) received a rating of serious in D2 due to recruitment from a small, convenience sample without clear inclusion procedures. Classen et al. (2001) received a rating of serious risk in D5 because participants who received either trauma-focused or present-focused therapy were combined into a single treatment group for analysis. For ROBINS-I ratings see Figures 5 and 6.

We conducted sensitivity analyses to examine the impact of removing studies rated at overall high risk of bias (RoB 2) or overall serious risk of bias (ROBINS-I). When all eligible studies were included (*k* = 37), the pooled effect was *g* = 0.63 (95% CI [0.40, 0.86]; *p* < .001). Excluding studies at overall high risk (RoB 2) and overall serious risk (ROBINS-I) yielded a similarly robust pooled effect (*g* = 0.63, 95% CI [0.36–0.90]; *p* < .001), which is the effect reported in the primary analyses.

### Effects of the Intervention

#### Synthesis of Results

##### Efficacy of Group-Based Treatment

A multilevel meta-analysis using a correlated and hierarchical effects model with robust variance estimation (CHE-RVE) was conducted to assess the effectiveness of group support interventions versus control or treatment-as-usual (TAU) conditions for reducing posttraumatic stress disorder (PTSD) symptoms. The analysis included 32 available effect sizes from 33 of the 40 included studies, where *n* is used to denote number of studies and *k* is used to denote number of studies included in subgroup analysis. We found that overall group-based interventions were significantly more effective than comparison conditions and produced a moderate effect size on PTSD symptom reduction (*k* = 32; *g* = 0.63; 95% *CI* [0.36, 0.90]; *p* < .001).

To assess the homogeneity of the effect sizes, we used the Q statistic. A significant Q indicates heterogeneity across studies and warrants further investigation. For between-group effects, Q was highly significant, *(Q* = 252.02, *df* = 31, *p* < .001, *I*^*2*^ *=* 87.6%). Variance decomposition showed that the majority of heterogeneity occurred at the between-study level (σ^2^ = 0.433), with no residual within-study variance detected. The *I*^*2*^ value is considerable based on threshold criteria in the Cochrane Handbook (<40 % low; 30–60 % moderate; 50–90 % substantial; 75–100 % considerable; Higgins & Green, 2008), thus providing support for our planned moderation analyses.

**Figure 7.**
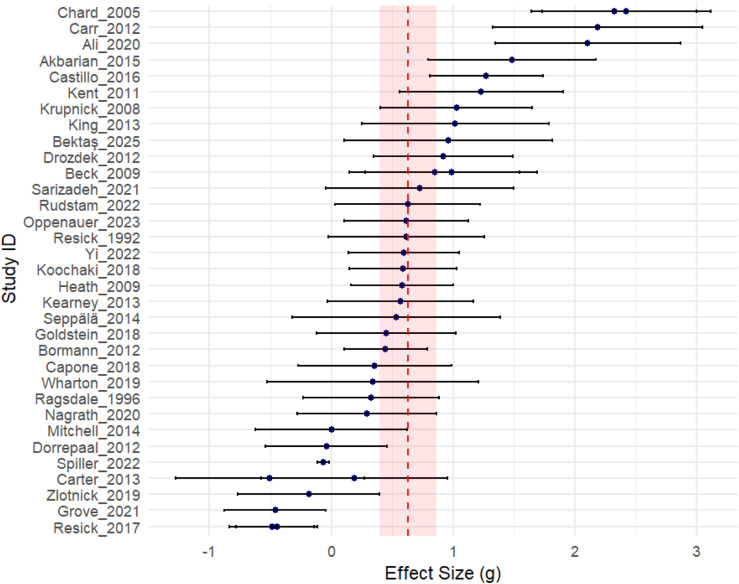
Forest Plot CHE-RVE Model

The forest plot derived from the CHE-RVE random-effects model (see Figure 7) indicates a statistically significant overall positive effect of the intervention, with a pooled effect size estimated around *g* = 0.4–0.5. This suggests that, on average, the intervention had a small to moderate positive impact across studies and their respective measures of PTSD symptoms. While the majority of studies reported positive effect sizes, a few (e.g., Grove et al., 2021; Resick et al., 2017) yielded near-zero or slightly negative results for certain group formats relative to active comparators. Notably, some studies (e.g., Carr et al., 2012; Chard, 2005) reported substantially larger effects, which may have influenced the pooled estimate.

#### Moderator Analyses

##### Shared Trauma Experience

Trauma similarity was a significant moderator, *QM*(3) = 28.33, *p* < .001. Interventions showed numerically larger effects for groups with shared trauma (*g* = 0.64, 95% CI [0.32, 0.95], *p* < .001) and groups with different trauma types (*g* = 0.84, 95% CI [0.36, 1.32], *p* < .001) than those with multiple trauma types (*g* = 0.32, 95% CI [−0.28, 0.91], *p* = .296). The Wald test was used to conduct head-to-head comparisons between subgroups, testing whether differences in effect sizes were statistically significant after accounting for clustering and correlations in the data. The Wald test for shared experience of trauma was non-significant indicating that when clustering was accounted for, the average effect across subgroups may be equivalent, *F* (2, 10.3) = 0.85, *p* = .454. Consistent with this result, pairwise comparisons indicated subgroup differences were not statistically significant from one another (shared vs different trauma: *F* (1, 13.2) = 0.42, *p* = .527; shared vs multiple trauma types: *F* (1, 6.44) = 1.02, *p* = .349; different vs multiple trauma types: *F* (1, 8.75) = 1.82, *p* = .211).

##### Stigmatised Trauma

Stigma was also a significant moderator, *QM*(2) = 15.82, *p* < .001. Interventions were more effective for groups where trauma is generally not considered stigmatised (e.g. motor vehicle accidents, natural disasters), (*g* = 0.70, 95% CI [0.19, 1.38], *p* = .044) compared to groups where trauma is generally stigmatised (e.g. sexual abuse, combat-related trauma), (*g* = 0.57, 95% CI [0.25, 0.90], *p* < .001). The Wald test produced non-significant results, *F* (1, 4.45) = 0.44, *p* = .541.

##### Intentionality of Trauma

Intentionality moderated treatment effects, *QM*(2) = 26.25, *p* < .001. Group interventions were effective for both intentional (*g* = 0.63, 95% CI [0.37, 0.89], *p* < .001) and unintentional traumas (*g* = 0.70, 95% CI [0.02, 1.39], *p* = .044). The Wald test was non-significant, *F* (1, 3.89) = 0.22, *p* = .666.

##### Gender Composition

Gender composition also moderated treatment effects, *QM*(3) = 29.65, *p* < .001. Women-only groups (*g* = 0.85, 95% CI [0.48, 1.22], *p* < .001) and mixed-gender groups (*g* = 0.50, 95% CI [0.15, .85], *p* = .005) produced larger effect sizes than men-only groups (*g* = 0.42, 95% CI [–0.28, 1.12], *p* = .243). The Wald test produced non-significant results, *F* (2, 8.56) = 1.12, *p* = .368. Consistent with the Wald test, pairwise comparisons indicated that subgroup differences were not statistically significant from one another (women-only vs men-only: *F* (1, 4.76) = 1.93, *p* = .226; women-only vs mixed-gender: *F* (1, 24.5) = 1.72, *p* = .202; men-only vs mixed-gender: *F* (1, 4.56) = 0.08, *p* = .795).

##### Comparator Conditions

Comparator type also significantly moderated treatment outcomes, *QM*(3) = 50.91, *p* < .001. The effect of comparator type was further supported by the Wald test which produced significant results, *F* (2, 4.95) = 7.98, *p* = .028. Pairwise comparisons among comparator types indicated that active controls differed significantly from waitlist and minimal attention controls, *F* (1, 2.53) = 17.10, *p* = .035, and waitlist and minimal attention controls differed significantly from TAU, *F* (1, 15.50) = 8.47, *p* = .010. No significant difference was observed between active controls and TAU, *F* (1, 3.37) = 2.59, *p* = .196. When comparing the intervention group directly to each comparator, a significant effect was observed relative to minimal attention and waitlist controls (*g* = 0.89, 95% CI [0.64, 1.14], *p* < .001), whereas effects against TAU (*g* = 0.28, 95% CI [-0.09, 0.65] *p* = .134) and active controls (*g* = −0.12, 95% CI [-0.74, 0.49], *p* = .691) were not significant. Relative to ISTSS clinical importance benchmarks, the pooled effect for comparisons against control groups (g = 0.89) exceeded the threshold for clinical relevance (>0.80). Effects for comparisons against treatment-as-usual (*g* = 0.28) and active controls (*g* = −0.12) did not reach the benchmark for clinical importance.

##### Group Size

A significant moderation effect was found for group size, *QM*(3) = 18.1, *p* < .001. Larger groups (8+ participants) produced stronger effects (*g* = 0.59, 95% CI [0.28, 0.90], *p* < .001) compared to smaller groups (6–8 participants: *g* = 0.39, *p* = .138; less than 6 participants: *g* = 0.44, *p* = .151). The Wald test produced non-significant results, *F* (2, 6.51) = 0.328, *p* = .731. Consistent with this result, pairwise comparisons indicated that subgroup differences were not statistically significant from one another (less than 6 vs 6–8 participants: *F* (1, 6.34) = 0.023, *p* = .885; less than 6 vs 8+ participants: *F* (1, 4.70) = 0.158, *p* = .709; 6–8 vs 8+ participants: *F* (1, 7.04) = 0.747, *p* = .416).

##### Outcome Measure

We examined whether the type of PTSD symptom measure moderated treatment outcomes. The model indicated significant residual heterogeneity, *QE* (33) = 203.16, *p* < .001, along with a statistically significant overall moderator effect, *QM*(4) = 31.63, *p* < .001. Individual estimates indicated that all PTSD measure types yielded significant effects. The IES-R was associated with the largest treatment effect, *g* = 1.02, 95% CI [0.47, 1.56], *p* < .001. This was followed by ‘other’ measures, that were used in less than three studies, (*g* = 0.60, 95% CI [0.26, .95], *p* < .001), PCL (*g* = 0.58, 95% CI [0.29, 0.87], *p* < .001), and CAPS (*g* = 0.57, 95% CI [0.21, 0.93], *p* = .002). These findings suggest that effect sizes were consistently moderate to large across different symptom measures, although variability in the magnitude of effect was present. The Wald-type test adjusted for small-sample degrees of freedom was not significant, *F* (3, 3.41) = 0.428, *p* = .746. Consistent with this result, pairwise comparisons indicated that outcome measures were not significantly different from one another (CAPS vs IES-R: *F* (1, 2.16) = 1.77, *p* = .307; CAPS vs Other: *F* (1, 4.94) = 0.022, *p* = .888; CAPS vs PCL: *F* (1, 7.16) = 0.002, *p* = .964; IES-R vs Other: *F* (1, 4.17) = 1.55, *p* = .279; IES-R vs PCL: *F* (1, 3.87) = 1.62, *p* = .274; Other vs PCL: *F* (1, 2.85) = 0.020, *p* = .898).

##### Treatment Type

A significant moderation effect was observed for treatment type, *QM*(6) = 34.65, *p* < .001. Group psychotherapy showed the largest effect (*k* = 5, *g* = 1.06, 95% CI [0.22, 1.28], *p* = .002), followed by alternative therapies (*k* = 7, *g* = 0.96, 95% CI [0.41, 1.51], *p* < .001) and group CBT (*k* = 7, *g* = 0.75, 95% CI [0.22, 1.28], *p* = .006). Other treatment types (e.g., movement-based, GCPT, and other group treatments) were not statistically significant. The Wald test also produced non-significant results, *F* (5, 10.6) = 1.56, *p* = .252. However, pairwise comparisons indicated that movement-based interventions produced significantly smaller effects than alternative therapies (*F* (1, 9.89) = 5.98, *p* = .035), with differences approaching significance for movement-based versus group psychotherapy (*F* (1, 6.55) = 4.25, *p* = .081) and other group interventions versus alternative therapies (*F* (1, 10.50) = 3.83, *p* = .077). All other pairwise contrasts were non-significant**.**

##### Age

Age was also a significant moderator, suggesting that older age was associated with greater effectiveness of group support in reducing PTSD symptoms, although the effect size was small (*g* = 0.01, 95% CI [0.01, 0.02], *p* < .001).

##### Social-Identity and Group-Based Factors

A key purpose of this review, as outlined in the protocol (Griffin et al., 2023), was to assess what social identity or group-based factors (if any) were measured and whether they were associated with the outcomes of interest. Of the included studies, only two explicitly measured group-based factors (Bormann et al., 2012; Heath, 2009). Bormann et al. (2012) measured participants’ identification with religious and or/spiritual groups, and Heath (2009) developed their own scale to assess the extent to which group facilitators worked to create a safe environment, appeared caring and compassionate, and managed crises or group conflict. Over half of studies (*n =* 22) referred to either social-identity or group-based factors in addition to their group-based treatment, but did not explicitly measure them, and less than half of studies (*n =* 16) did not refer to or measure any social-identity or group-based factors. Examples of social-identity and group-based factors referred to, but not measured by studies, are presented in Table 3.

#### Publication Bias

**Figure 8.**
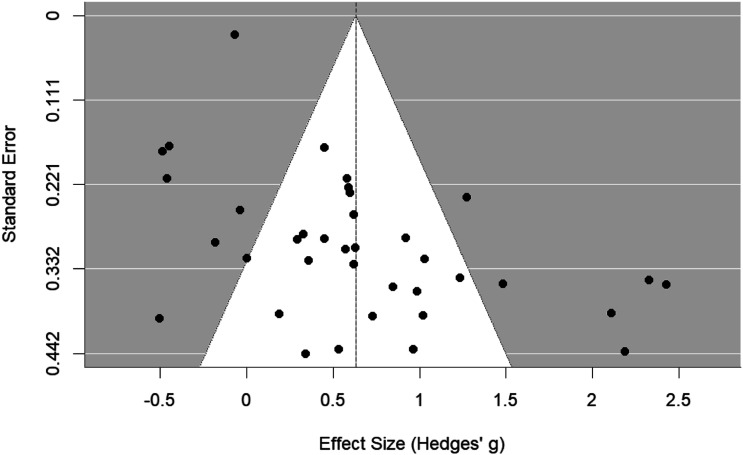
Funnel Plot CHE-RVE Model

The asymmetry observed in the funnel plot (Figure 8) suggested possible publication bias in that small or negative effects may be underreported. These observations were supported by the Egger’s test which was statistically significant (*p* < .001).

#### Secondary outcomes

Of the secondary outcomes pre-specified in our protocol (depression, psychosomatic symptoms, and post-traumatic growth), only depression was reported in enough studies to allow for synthesis of findings. Of the 40 included studies, 19 measured depression in addition to PTSD. Among these, 9 studies reported significant reductions in depressive symptoms in treatment groups post-intervention, including cognitive-behavioral approaches (e.g., GCBT, GCPT, and REBT), as well as in other structured group therapies such as music therapy, interpersonal psychotherapy, and resilience-oriented programs. In some cases, the effectiveness of the treatment group in reducing depressive symptoms was also observed at 3- and 6-month follow-up (e.g., Resick & Schnicke, 1992; Yi et al., 2022). Six studies reported reductions in depressive symptoms in both treatment and control conditions (e.g., waitlist, treatment-as-usual). These results suggest that while group-based PTSD interventions primarily target PTSD symptoms, they may also confer modest benefits for comorbid depressive symptoms.

#### Adverse Effects

Of the 40 included studies, only 10 reported information relevant to adverse effects. Two studies explicitly stated that no adverse effects occurred. Five studies reported varying degrees of adverse effects, ranging from mild psychological discomfort, such as brief traumatic memories during yoga or mindfulness exercises, temporary increases in anxiety, or distress triggered by music, to more overt adverse reactions. Resick et al. (2017) noted 17 psychological events judged by participants to be possibly related to trauma-focused interventions. These events occurred because of increased symptoms evoked by baseline assessment procedures (4 patients) or the trauma focus of therapy (7 patients in group CPT and 6 patients in individual CPT). Three studies did not report specific adverse effects but raised some concerns, one noted that some program graduates experienced a sense of letdown after leaving the program (Ragsdale et al., 1996), another highlighted the importance of facilitator supervision to reduce risk of secondary trauma (Kara et al., 2025), and a third concluded that group therapy is not appropriate for all individuals (Hetz, 1999). Overall, serious adverse effects were rare, but the limited and inconsistent reporting underscores the need for further assessment and reporting of adverse effects in group-based PTSD interventions.

## Discussion

### Summary of Main Results

The present review found that group-based treatments were generally effective in reducing symptoms of PTSD with outcomes consistent with previous research (Schwartze et al., 2019; Sloan et al., 2013). Our findings revealed that the following treatments yielded the largest effects on PTSD symptoms; group psychotherapy, group CBT, and alternative treatments (e.g. mindfulness-based). On average, individuals receiving group-based treatments experienced large improvements in PTSD symptoms compared to controls (i.e., minimal attention groups or waitlist controls). When interpreted relative to ISTSS clinical importance benchmarks, the pooled effect for comparisons against control groups exceeded the threshold for clinical relevance, whereas effects for comparisons against treatment-as-usual and active controls did not meet clinical criteria. Although comparisons with active controls (e.g. the same treatment in individual format) showed slightly better outcomes than group-based approaches, the difference was not statistically significant, suggesting that both formats can offer comparable benefits depending on context and available resources.

In line with the social identity approach (Tajfel & Turner, 1979; Turner et al., 1987), certain group characteristics were associated with variation in effect sizes. While several moderators were significant in conventional omnibus QM tests, cluster-robust variance estimation (RVE) with small-sample correction indicated that some subgroup differences were not statistically significant. This is typical of conservative tests applied to small samples and should therefore be interpreted with caution. Larger groups (more than eight participants) showed larger effect sizes, which extends previous research suggesting an optimal group size of 6–8 participants (Yalom, 1995). We also explored if gender composition would have an effect as we expected groups comprised of individuals from the same gender might share more relevant social identities (S. A. Haslam et al., 2012; Muldoon, Haslam, et al., 2019). We found that effects were strongest for groups that included all women. Indeed, this is consistent with past reviews (Schwartze et al., 2019; Sloan et al., 2013). However, it is worth noting (and acknowledging the small number of studies) that mixed gender groups showed larger effect sizes than men-only groups. Differences in effect size magnitude may reflect differences in group dynamics across gender compositions (Murillo Parra & Nadal, 2017), as well as the possibility that the effectiveness of group composition depends on the nature of the trauma being addressed. Given that trauma exposure is strongly patterned by gender (Muldoon, 2024), gender and trauma type are likely to interact, such that the fit between group composition and trauma characteristics may influence intervention effectiveness. Further research is therefore needed to examine these interactions and clarify how gender composition and trauma type jointly shape group-based intervention outcomes.

Groups were effective whether participants shared the same trauma or had different trauma experiences, but effect sizes were smaller for groups involving multiple traumatic events. Individuals who have experienced multiple or chronic traumas often present with a distinct, more complex clinical profile than those exposed to single traumatic events (Kube et al., 2023). While both can lead to PTSD, repeated or prolonged trauma frequently results in Complex PTSD (C-PTSD). Taken together, these factors are likely to render the treatment of these cohorts significantly more challenging. We had hypothesised effects might be stronger in groups where individuals all experienced the same trauma as this might foster a greater sense of belonging, shared experience, and/or social identification (Acharya & Muldoon, 2017; Drury, 2012; Muldoon, Haslam, et al., 2019), however from the results it appears that group-based interventions were comparably effective in each type of setting. Further, effect sizes were larger for non-intentional traumas, this is in line with our predictions as trauma, especially that caused intentionally by others, can influence trust in others and the ability to bond with others which might limit the effectiveness of the group (Charuvastra & Cloitre, 2008).

We also examined whether the type of group intervention influenced outcomes. Moderator analyses suggested variation across treatment types with cognitive behavioural therapy (CBT), group psychotherapy, and alternative approaches such as mindfulness or music therapy demonstrating larger effect sizes relative to group cognitive processing therapy (CPT), yoga, or mixed-approach therapies like Dialectical Behavior Therapy (DBT) and Acceptance and Commitment Therapy (ACT). However, follow-up more conservative head-to-head comparisons indicated that while movement-based interventions produced significantly smaller effects than alternative therapies (e.g. mindfulness based, music therapy), other comparisons were not statistically significant. These findings suggest that although some intervention types appeared to produce larger effects, most treatment modalities performed comparably overall.

Finally, although many studies mentioned group dynamics or social identity, very few measured these factors directly, highlighting a key gap in our understanding of how group processes contribute to treatment outcomes.

### Overall Completeness and Applicability of Evidence

Overall, our findings provide support for the effectiveness of group-based treatments in improving symptoms of PTSD, specifically group psychotherapy, group CBT and alternative treatments (e.g. mindfulness-based) produced a larger effect on PTSD symptoms compared to controls and TAU conditions. However, it is important to note that most of the included studies were conducted in the USA on people who had experienced combat-related trauma and therefore findings may not extend to other settings and contexts. Men-only groups were under-represented compared to women-only and mixed groups, and the mean age of participants across groups was 43.3 years, therefore findings may differ for men and younger samples. The included studies represent a broad range of treatment types, with some producing larger effects than others.

We intended to examine effects of group-based interventions on post-traumatic growth (PTG), however, none of the studies in this review measured PTG. We feel this is important to consider in future work as groups may not only reduce PTSD symptoms, but may also foster PTG, and these constructs should be measured independently (Capaldi et al., 2024).

We also intended to measure the effects of group-based and social processes on our primary outcome, PTSD symptoms. However, despite 24 studies referring to group and/or social processes in their methodology (see Table 3), only two of these studies explicitly measured these processes (Bormann et al., 2012; Heath, 2009). Due to this, we were unable to address our final objective of the review. Both Beck et al. (2009) and Carr et al. (2012) acknowledged that outcomes could have been achieved because of a ‘group-effect’ rather than a specific treatment effect, however, neither study included any group-based measures. These findings also highlight the fact that group-based interventions for people with PTSD lack a conceptual framework for understanding the role of underlying social processes and how they might contribute to the effectiveness of group-based interventions. Based on the group-based nature of this work, we feel it is imperative that these underlying processes are interrogated further in order to realise their potential in harnessing group support.

### Quality of the Evidence

Considerable heterogeneity was observed across studies (*Q* = 252.02, *df* = 31, *p* < .001; *I*^2^ = 87.6%), driven almost entirely by between-study differences (σ^2^ = 0.433). This variability largely reflects differences between studies rather than measurement error, likely due to variations in populations, intervention characteristics, and contextual factors. Moderator analyses indicated that comparator type, trauma characteristics, gender composition, group size, treatment type, and outcome measure partially explained heterogeneity. However, substantial residual variance remained, suggesting additional unmeasured factors (e.g., facilitator expertise, cultural context, or group processes) also contribute to differences in effect size. These findings highlight that while group-based PTSD interventions are generally effective, the magnitude of effect may vary across populations, interventions, and study designs.

Most studies were rated as moderate risk of bias. Given the practical challenges associated with conducting RCTs, such as recruiting and retaining people with PTSD for group-based interventions, moderate levels of bias would be expected. Many non-RCTs were also included and therefore bias due to non-randomisation of participants to treatment and control conditions was also an issue. The lack of active controls also limits the extent to which we can compare group interventions to other forms of treatment (e.g. same treatment in an individual format). Small sample sizes and limited generalisability of findings were reported in nearly all of the studies included in our review, again this is due to the nature of participants’ diagnosis and the specific contexts in which group-based interventions are offered. This also meant that few studies conducted any follow-up analysis, so less is known about the long-term impact of group-based treatments on PTSD symptoms. Studies also referred to the heterogeneity in participants’ treatment histories, as well as differences in their duration and severity of PTSD symptoms which may have contributed to bias in results (Dorrepaal et al., 2012).

### Potential Biases in the Review Process

The review has both strengths and limitations. We are confident that our review of the published literature is inclusive and up to date. We made efforts to identify grey literature by scrutinizing citations of public works, by using a call for research following the publication of our protocol, as well as by contacting researchers in the field. Clearly, we were restricted by the available studies which were disproportionately conducted in the USA and with military veterans. Fewer studies oriented to the effect of group-based treatments on people affected by trauma in civilian life, and very few studies from non-WEIRD (Western, Educated, Industrialized, Rich, Democratic) contexts were available (Henrich et al., 2010). Due to the number of studies available, we categorized treatments into group-based approaches and comparator conditions for the purposes of analysis. We categorized interventions into ‘alternative’ and ‘movement-based’ groups for the purposes of analysis. However, the treatment approaches within each category were heterogeneous and not fully equivalent. Equally, treatment approaches that were only evaluated in one or two of our reviewed studies were considered as ‘other’. This category of treatment approaches was, however, reasonably heterogenous. Notably, although we had a clear interest in identifying the group-based processes that may be associated with any group-based treatment effect, available studies did not allow us to evaluate this evidence. Finally, while studies in English were of primary interest we also allowed for any articles with an English translation available to be included. This may have led to the exclusion of relevant non-English studies, which could limit the generalisability of our findings.

### Agreements and Disagreements With Other Studies or Reviews

The present review addresses a key gap concerning the effectiveness of *group*-based treatments for PTSD (Cusack et al., 2016; Lewis et al., 2020). Our findings support the broader consensus that group-based interventions are effective, showing large improvements compared to control conditions, consistent with prior meta-analyses (e.g., Barrera et al., 2013; Schwartze et al., 2019). When compared to treatment-as-usual, effects were moderate but not statistically significant, aligning with Sloan et al. (2013) who reported limited advantages over active comparators. The finding that individual treatments produced greater, though non-significant, effects than group-based interventions is reflected in prior work (Bisson et al., 2013; Schwartze et al., 2019; Sloan et al., 2013) where comparable outcomes across individual and group formats were reported.

We found variation in outcomes based on treatment modality, with group CBT and alternative therapies (e.g., mindfulness-based, music therapy) demonstrating larger pooled effects than group CPT and movement-based or integrative approaches. This contrasts with previous work (Lewis et al., 2020; Resick et al., 2017) reporting strong outcomes for CPT, particularly in individual treatment formats. The discrepancy may relate to population characteristics (e.g., active-duty military), intervention length, facilitator training, or group process factors not captured consistently across studies.

We further identified underexplored variables influencing treatment efficacy. Women-only groups demonstrated larger effects than men-only groups and larger group sizes (>8 participants) were associated with stronger effect sizes; however, these subgroup differences were non-significant in more conservative head-to-head comparisons. Additionally, reduced efficacy among participants with multiple trauma exposures was consistent with Bradley et al.'s (2005) concerns about limited generalizability in complex PTSD cases.

## Authors’ Conclusions

### Implications for Practice and Policy

Based on our review of the available research, group-based treatments were effective in reducing PTSD symptoms, with group psychotherapy, group CBT, and alternative treatments producing the largest effects. For this reason, group-based treatments are an important approach to tackling PTSD, particularly in services where resources are limited, as group formats allow multiple participants to be treated simultaneously and may provide a more efficient and cost-effective option in clinical practice. Group-based treatments produced significant and large effects compared to controls (minimal attention and waitlist controls). Effects were non-significant compared to active controls and treatment as usual, suggesting that group formats may offer broadly comparable benefits. However, results should be interpreted with caution, given the relatively small number of studies and limited sample sizes typical of PTSD research.

Whilst these findings offer an evidence base for this cost-effective approach in pressed mental health services, it is important to be mindful of the parameters where this approach is most likely to be of benefit. First, group size appeared to have a positive effect on treatment outcomes. Larger groups with more than eight participants showed descriptively larger effects on PTSD symptoms. Similarly, groups in which participants shared trauma experiences and women-only groups demonstrated stronger effect sizes. It is worth noting that while these findings should be interpreted cautiously given non-significant follow-up head-to-head comparisons, the small number of studies included limited statistical power to detect effects should they exist. More research is needed to elucidate how group size, and group gender composition, may affect group treatment outcomes for PTSD. It may be the case that larger groups, and women-only groups may allow for a sense of a collective self, arising from a shared experience of trauma, the limited number of studies available have not allowed us to conclude this.

Based on these findings, facilitators may consider forming larger groups (more than eight participants), grouping participants with similar trauma experiences, and using women-only or mixed-gender groups to support stronger treatment outcomes. Facilitators could also explicitly orient participants to shared experiences, emphasizing that although trauma histories may differ, individuals are not alone and can recognize common understanding, helping to normalize experiences and foster mutual support (Muldoon et al., 2025).

### Implications for Research

Future research should prioritize expanding the evidence base beyond predominantly U.S.-based, military-focused samples to include more diverse populations and trauma types. Although group-based interventions were generally effective, considerable heterogeneity across studies indicates that outcomes vary depending on population characteristics, group composition, intervention type, and study context, highlighting the need for more nuanced research. To better account for this between-study variability, future work should aim for greater standardisation of outcome reporting, improved measurement of group processes and social-identity mechanisms, and more consistent reporting of comparator conditions and adverse effects. Studies should also investigate why certain group modalities (e.g., mindfulness-based approaches) produced larger effects compared to movement-based interventions, potentially through more detailed analysis of treatment components, facilitator training, and delivery context.

Critically, social identity and group-based mechanisms remain underexplored, despite theoretical support for their role in enhancing treatment outcomes. Only two studies in our review explicitly measured these constructs, limiting insight into how group cohesion, identification, or interpersonal dynamics influence effectiveness. Given growing evidence that social identification-building interventions improve health outcomes (Steffens et al., 2021) and research demonstrating group processes (e.g., social support, cohesion, social norms) affect physiological indices of stress (Lebedová et al., 2026), future trials should incorporate validated measures of group processes to test these mechanisms directly.

Additionally, moderators such as group size, gender composition, and trauma homogeneity warrant systematic exploration through pre-registered, well-powered clinical trials. Finally, research should address methodological gaps noted in previous reviews, including limited long-term follow-up and the need to investigate how complex trauma may influence outcomes, to inform best practices for PTSD treatment.

## Supplemental Material

Supplemental Material - Group-Based Interventions for Posttraumatic Stress Disorder: A Systematic Review and Meta-Analysis of the Role of Trauma Type Supplemental Material for Group-Based Interventions for Posttraumatic Stress Disorder: A Systematic Review and Meta-Analysis of the Role of Trauma Type by Éadaoin Whelan, Siobhán M. Griffin, Elayne Ahern, Alžběta Lebedová, Grace McMahon, Daragh Bradshaw, Orla T, Muldoon in Campbell Systematic Reviews

## Data Availability

The data that support the findings of this study are available from the corresponding author upon reasonable request.*
