# Supplementary material for: Group-Based Interventions for Posttraumatic Stress Disorder: A Systematic Review and Meta-Analysis of the Role of Trauma Type
Source: Campbell Syst Rev. 2026 Jul 30;22(3):18911803261471160. doi: 10.1177/18911803261471160 (PMC13424537; doi:10.1177/18911803261471160)
Supplement: Supplemental Material - Group-Based Interventions for Posttraumatic Stress Disorder: A Systematic Review and Meta-Analysis of the Role of Trauma Type [file sj-pdf-1-cam-10.1177_18911803261471160.pdf]

Appendices

Table 1A

Criteria used to determine eligibility

Inclusion Criteria
▪ At least 70% of the sample must have been diagnosed with PTSD, screened positively for PTSD, or referred for treatment by a medical professional for their PTSD symptoms.<br><br>▪ Group-based intervention with a minimum of 3 people per group, and a minimum of 5 sessions.<br><br>▪ Primary aim of intervention must be to reduce symptoms of PTSD.<br><br>▪ There is one group of participants undergoing a *group-based* treatment for PTSD and another cohort of people either in a control/no treatment/treatment as usual group or doing a similar treatment, but in an individual format (e.g., group-based CBT group and an individual based CBT comparator).<br><br>▪ PTSD must be assessed before and after the intervention.<br><br>▪ Participants can be taking medication for PTSD but must not be engaging in other therapies outside of the intervention – other than "treatment as usual" or "usual care".<br><br>▪ Participants must *all* be >18 years of age (i.e., adults).

Table 2A

Sample search strategy for Ovid MEDLINE searches

1	exp Stress Disorders, Post-Traumatic/
2	(PTSD or ((posttrauma* or post-trauma* or post trauma*) adj3 (stress* or disorder* or psych* or symptom? or Neuroses)) or acute stress disorder* or combat disorder* or war neuros*).ti,ab,kf.
3	exp Stress Disorders, Post-Traumatic/
4	(group* adj5 (treat* or intervention* or therap* or train* or program* or session* or setting* or factor* or process* or counsel* or psychotherap* or psychoeducat* or psycho-educat*)).mp.
5	Controlled clinical trial.pt.
6	Randomi#ed controlled trial.pt.

7	(RCT or at random or (random* adj3 (assign* or allocat* or control* or crossover or cross-over or design* or divide* or division or number))).ti,ab,kf.
8	trial.ab,ti,kf.
9	(control* and (trial or study or group*) and (placebo or waitlist* or wait* list* or ((treatment or care) adj2 usual))).ti,ab,kf,hw.
10	exp cohort studies/ or exp epidemiologic studies/ or exp clinical trial/ or exp evaluation studies as topic/ or exp statistics as topic/
11	((control and (group* or study or trial)) or (time and factors) or program or survey* or ci or cohort or comparative stud* or evaluation studies or follow-up*).mp.
12	or/1-2
13	or/3-4
14	or/5-11
15	12 and 13 and 14
16	animals/ not humans/
17	15 not 16
18	limit 17 to (english language and "all adult (19 plus years)")

Table 3A

Tailored Search Strategy for PsychINFO

1	DE "Posttraumatic Stress Disorder" OR DE "Complex PTSD" OR DE "DESNOS"
2	TI (PTSD OR ((posttrauma* OR post-trauma* OR post trauma*) NEAR/3 (stress* OR disorder* OR psych* OR symptom? OR Neuroses)) OR acute stress disorder* OR combat disorder* OR war neuros*)
3	AB (PTSD OR ((posttrauma* OR post-trauma* OR post trauma*) NEAR/3 (stress* OR disorder* OR psych* OR symptom? OR Neuroses)) OR acute stress disorder* OR combat disorder* OR war neuros*)
4	KW (PTSD OR ((posttrauma* OR post-trauma* OR post trauma*) NEAR/3 (stress* OR disorder* OR psych* OR symptom? OR Neuroses)) OR acute stress disorder* OR combat disorder* OR war neuros*)
5	DE "Group Psychotherapy" OR DE "Encounter Group Therapy" OR DE "Therapeutic Community"
6	TI (group* NEAR/5 (treat* OR intervention* OR therap* OR train* OR program*

	OR session* OR setting* OR factor* OR process* OR counsel* OR psychotherap* OR psychoeducat* OR psycho-educat*))
7	AB (group* NEAR/5 (treat* OR intervention* OR therap* OR train* OR program* OR session* OR setting* OR factor* OR process* OR counsel* OR psychotherap* OR psychoeducat* OR psycho-educat*))
8	KW (group* NEAR/5 (treat* OR intervention* OR therap* OR train* OR program* OR session* OR setting* OR factor* OR process* OR counsel* OR psychotherap* OR psychoeducat* OR psycho-educat*))
9	PT (Controlled clinical trial)
10	PT (Randomi#ed controlled trial)
11	TI ((RCT OR at random OR (random* NEAR/3 (assign* OR allocat* OR control* OR crossover OR cross-over OR design* OR divide* OR division OR number))))
12	AB ((RCT OR at random OR (random* NEAR/3 (assign* OR allocat* OR control* OR crossover OR cross-over OR design* OR divide* OR division OR number))))
13	KW ((RCT OR at random OR (random* NEAR/3 (assign* OR allocat* OR control* OR crossover OR cross-over OR design* OR divide* OR division OR number))))
14	TI (trial)
15	AB (trial)
16	KW (trial)
17	TI ((control* AND (trial OR study OR group*) AND (placebo OR waitlist* OR wait* list* OR ((treatment OR care) N2 usual))))
18	AB ((control* AND (trial OR study OR group*) AND (placebo OR waitlist* OR wait* list* OR ((treatment OR care) N2 usual))))
19	KW ((control* AND (trial OR study OR group*) AND (placebo OR waitlist* OR wait* list* OR ((treatment OR care) N2 usual))))
20	(DE "Cohort Analysis") OR (DE "Epidemiology")) OR (DE "Clinical Trials")) OR (DE "Evaluation")) OR (DE "Statistics")
21	TI (((control AND (group* OR study OR trial)) OR (time AND factors) OR program OR survey* OR ci OR cohort OR comparative stud* OR evaluation studies OR follow-up*))
22	AB (((control AND (group* OR study OR trial)) OR (time AND factors) OR program OR survey* OR ci OR cohort OR comparative stud* OR evaluation studies OR follow-up*))
23	KW (((control AND (group* OR study OR trial)) OR (time AND factors) OR program OR survey* OR ci OR cohort OR comparative stud* OR evaluation studies OR follow-up*))
24	(S1 OR S2 OR S3 OR S4)
25	(S5 OR S6 OR S7 OR S8)
26	(S9 OR S10 OR S11 OR S12 OR S13 OR S14 OR S15 OR S16 OR S17 OR S18 OR S19 OR S20 OR S21 OR S22 OR S23)
27	(S24 AND S25 AND S26)
28	(animals/ not humans/)
29	(S27 NOT S28)
30	limit S29 to ("English" AND ("Age Groups:Adulthood (18 yrs & older)")

Table 4A

Tailored Search for the Cochrane Database

1	MeSH descriptor: [Stress Disorders, Post-Traumatic] explode all trees
2	(PTSD OR ((posttrauma* OR post-trauma* OR post NEXT trauma*) NEAR/3 (stress* OR disorder* OR psych* OR symptom? OR Neuroses)) OR acute NEXT stress NEXT disorder* OR combat NEXT disorder* OR war NEXT neuros*):ti,ab,kw
3	MeSH descriptor: [Psychotherapy, Group] explode all trees
4	MeSH descriptor: [Group Processes] explode all trees
5	(group* NEAR/5 (treat* OR intervention* OR therap* OR train* OR program* OR session* OR setting* OR factor* OR process* OR counsel* OR psychotherap* OR psychoeducat* OR psycho-educat*)):ti,ab,kw
6	Controlled clinical trial:pt
7	Randomi?ed controlled trial:pt
8	((RCT OR at random OR (random* NEAR/3 (assign* OR allocat* OR control* OR crossover OR cross-over OR design* OR divide* OR division OR number)))):ti,ab,kw
9	trial:ti,ab,kw
10	((control* AND (trial OR study OR group*) AND (placebo OR waitlist* OR wait* NEXT list* OR ((treatment OR care) N2 usual)))):ti,ab,kw
11	MeSH descriptor: [Cohort Studies] explode all trees
12	MeSH descriptor: [Epidemiologic Studies] explode all trees
13	MeSH descriptor: [Clinical Trial] explode all trees
14	MeSH descriptor: [Statistics] explode all trees
15	(((control AND (group* OR study OR trial)) OR (time AND factors) OR program OR survey* OR ci OR cohort OR comparative stud* OR evaluation studies OR follow-up*)):ti,ab,kw
16	#1 OR #2
17	{OR #3-#5}
18	{OR #6-#15}
19	#16 AND #17 AND #18
20	MeSH descriptor: [Animals] explode all trees
21	#19 NOT #20

Table 5A

Tailored Search for Embase database

1	'posttraumatic stress disorder'/exp
2	'ptsd':ti,ab,kw OR ((('posttrauma*' OR 'post-trauma*' OR 'post trauma*') NEAR/3 ('stress*' OR 'disorder*' OR 'psych*' OR 'symptom?' OR 'neuroses')):ti,ab,kw) OR 'acute stress disorder*':ti,ab,kw OR 'combat disorder*':ti,ab,kw OR 'war neuros*':ti,ab,kw
3	'group therapy'/exp
4	'group process'/exp
5	('group*' NEAR/5 ('treat*' OR 'intervention*' OR 'therap*' OR 'train*' OR 'program*' OR 'session*' OR 'setting*' OR 'factor*' OR 'process*' OR 'counsel*' OR 'psychotherap*' OR 'psychoeducat*' OR 'psycho-educat*')):ti,ab,kw

6	'controlled clinical trial'/de
7	'randomized controlled trial'/de
8	'rct':ti,ab,kw OR 'at random':ti,ab,kw OR (('random*' NEAR/3 ('assign*' OR 'allocat*' OR 'control*' OR 'crossover' OR 'cross-over' OR 'design*' OR 'divide*' OR 'division' OR 'number')):ti,ab,kw)
9	trial:ti,ab,kw
10	'control*':ti,ab,kw AND ('trial':ti,ab,kw OR 'study':ti,ab,kw OR 'group*':ti,ab,kw) AND ('placebo':ti,ab,kw OR 'waitlist*':ti,ab,kw OR 'wait* list*':ti,ab,kw OR ((('treatment' OR 'care') NEAR/2 'usual'):ti,ab,kw))
11	'cohort analysis'/exp OR 'epidemiology'/exp OR 'clinical trial'/exp OR 'evaluation study'/mj OR 'statistics'/mj
12	((‘control’ and (‘group*’ or ‘study’ or ‘trial’)) or (‘time’ and ‘factors’) or ‘program’ or ‘survey*’ or ‘ci’ or ‘cohort’ or ‘comparative stud*’ or ‘evaluation studies’ or ‘follow-up*’):ti,ab,kw
13	#1 OR #2
14	#3 OR #4 OR #5
15	#6 OR #7 OR #8 OR #9 OR #10 OR #11 OR #12
16	#13 AND #14 AND #15
17	'animal experiment'/de
18	#16 NOT #17
19	#18 AND ([adult]/lim OR [aged]/lim OR [very elderly]/lim) AND [english]/lim

Table 6A

Search Strategy tailored for PTSDPubs

1	[MAINSUBJECT.EXACT.EXPLODE] ("PTSD") OR (((TIABSU("PTSD" OR (("posttrauma*" OR "post-trauma*" OR "post trauma*") NEAR/3 ("stress*" OR "disorder*" OR "psych*" OR "symptom?" OR "Neuroses")) OR "acute stress disorder*" OR "combat disorder*" OR "war neuros*"))))
2	[MAINSUBJECT.EXACT.EXPLODE] ("Group Psychotherapy") OR (((TIABSU (“group*” NEAR/5 (“treat*” or “intervention*” or “therap*” or “train*” or “program*” or “session*” or “setting*” or “factor*” or “process*” or “counsel*” or “psychotherap*” or “psychoeducat*” or “psycho-educat*”)))))
3	[MAINSUBJECT.EXACT.EXPLODE] ("Epidemiology") OR [MAINSUBJECT.EXACT.EXPLODE] ("Randomized Clinical Trial") OR [MAINSUBJECT.EXACT.EXPLODE] ("Clinical Trial") OR [MAINSUBJECT.EXACT]("Program Evaluation") OR [MAINSUBJECT.EXACT]("Statistical Analysis") OR (TIABSU(“Controlled clinical trial” OR “Randomi?ed controlled trial”)) OR (TIABSU(“trial” OR “RCT” or “at random” or (random* NEAR/3 (assign* or allocat* or control* or crossover or cross-over or design* or divide* or division or number)))) OR (TIABSU(control* and (“trial” or “study” or “group*”) and (“placebo” or “waitlist*” or “wait* list*” or ((“treatment” or “care”) NEAR/2 “usual”)))) OR (TIABSU((“control” and (“group*” or “study” or “trial”)) or (“time” and “factors”) or “program” or “survey*” or “ci” or “cohort” or “comparative stud*” or “evaluation studies” or “follow-up*”))

4	Limit English

Table 7A

Search Strategy tailored for ASSIA

1	MAINSUBJECT.EXACT("Acute stress disorder") OR MAINSUBJECT.EXACT.EXPLODE("Posttraumatic stress disorder") OR (((TIABSU("PTSD" OR (("posttrauma*" OR "post-trauma*" OR "post trauma*") NEAR/3 ("stress*" OR "disorder*" OR "psych*" OR "symptom?" OR "Neuroses")) OR "acute stress disorder*" OR "combat disorder*" OR "war neuros*"))))
2	MAINSUBJECT.EXACT.EXPLODE("Group therapy") OR MAINSUBJECT.EXACT.EXPLODE("Group processes") OR (((TIABSU (“group*” NEAR/5 (“treat*” or “intervention*” or “therap*” or “train*” or “program*” or “session*” or “setting*” or “factor*” or “process*” or “counsel*” or “psychotherap*” or “psychoeducat*” or “psycho-educat*”)))))
3	MAINSUBJECT.EXACT.EXPLODE("Epidemiology") OR MAINSUBJECT.EXACT.EXPLODE("Randomized controlled trials") OR MAINSUBJECT.EXACT.EXPLODE("Clinical trials") OR MAINSUBJECT.EXACT("Evaluation") OR MAINSUBJECT.EXACT("Statistics") OR(TIABSU(“Controlled clinical trial” OR “Randomi?ed controlled trial”)) OR (TIABSU(“trial” OR “RCT” or “at random” or (“random*” NEAR/3 (“assign*” or “allocat*” or “control*” or “crossover” or “cross-over” or “design*” or “divide*” or “division” or “number”)))) OR (TIABSU(“control*” and (“trial” or “study” or “group*”) and (“placebo” or “waitlist*” or “wait* list*” or ((“treatment” or “care”) NEAR/2 “usual”)))) OR (TIABSU((“control” and (“group*” or “study” or “trial”)) or (“time” and “factors”) or “program” or “survey*” or “ci” or “cohort” or “comparative stud*” or “evaluation studies” or “follow-up*”))
4	Limit English

Table 8A

Search Strategy for Proquest Dissertations

1	((TIABSU("PTSD" OR (("posttrauma*" OR "post-trauma*" OR "post trauma*") NEAR/3 ("stress*" OR "disorder*" OR "psych*" OR "symptom?" OR "Neuroses")) OR "acute stress disorder*" OR "combat disorder*" OR "war neuros*")))
2	TIABSU("group*" NEAR/5 ("treat*" OR "intervention*" OR "therap*" OR "train*" OR "program*" OR "session*" OR "setting*" OR "factor*" OR "process*" OR "counsel*" OR "psychotherap*" OR "psychoeducat*" OR "psycho-educat*"))
3	TIABSU(“Controlled clinical trial*” OR “Randomi?ed controlled trial” OR “clinical trial*” OR “evaluation”) OR (TIABSU(“trial” OR “RCT” or “at random”

	or (“random*” NEAR/3 (“assign*” or “allocat*” or “control*” or “crossover” or “cross-over” or “design*” or “divide*” or “division” or “number”)))) OR (TIABSU(“control*” and (“trial” or “study” or “group*”) and (“placebo” or “waitlist*” or “wait* list*” or ((“treatment” or “care”) NEAR/2 “usual”)))) OR (TIABSU((“control” and (“group*” or “study” or “trial”)) or (“time” and “factors”) or “program” or “survey*” or “ci” or “cohort” or “comparative stud*” or “evaluation studies” or “follow-up*”))
4	Limit English

Other Searches Completed

Search terms for PsyArXiv and MedRXiv

(“PTSD” OR “posttrauma* stress” OR “posttrauma* disorder” OR “posttrauma* symptom?” OR “posttrauma* neuroses” OR “post-trauma* stress” OR “post-trauma* disorder” OR “post-trauma* symptom?” OR “post-trauma* neuroses” OR “post trauma* stress” OR “post trauma* disorder” OR “post trauma* symptom?” OR “post trauma* neuroses” OR "acute stress disorder*" OR "combat disorder*" OR "war neuros*") AND (“group* treat*” OR “group* intervention*” OR “group* therap*” OR “group* train*” OR “group* program*” OR “group* session*” OR “group* setting*” OR “group* factor*” OR “group* process*” OR “group* counsel*” OR “group* psychotherapy*” OR “group* psychoeducat*” OR “group* psycho-educat*”) AND (“controlled clinical trial*” OR “Randomi?ed controlled trial” OR “clinical trial*” OR “evaluation” OR “RCT” or “at random” or “crossover design” or “cross-over design” OR “control* trial” OR “control study” OR “control group” OR “placebo” or “waitlist*” or “wait* list*” or “treatment as usual” or “care as usual” or “control cohort” or “comparative stud*” or “evaluation studies” or “follow-up*”)

Handsearching the European Journal of Psychotraumatology

The below search terms were run twice, firstly with the 'title' filter, and subsequently with the 'abstract' filter'

[[Publication Title: "ptsd"] OR [Publication Title: "posttraumatic stress"] OR [Publication Title: "posttraumatic disorder"] OR [Publication Title: "posttraumatic symptom"] OR [Publication Title: "posttraumatic neuroses"] OR [Publication Title: "post-traumatic stress"] OR [Publication Title: "post-traumatic disorder"] OR [Publication Title: "post-traumatic symptom"] OR [Publication Title: "post-traumatic neuroses"] OR [Publication Title: "post traumatic stress"] OR [Publication Title: "post traumatic disorder"] OR [Publication Title: "post traumatic symptom"] OR [Publication Title: "post traumatic neuroses"] OR [Publication Title: "acute stress disorder"] OR [Publication Title: "combat disorder"] OR [Publication Title: "war neuroses"]] AND [[Publication Title: "group treatment"] OR [Publication Title: "group intervention"] OR [Publication Title: "group therapy"] OR [Publication Title: "group training"] OR [Publication Title: "group program"] OR [Publication Title: "group session"] OR [Publication Title: "group setting"] OR [Publication Title: "group factor"] OR [Publication Title: "group process"] OR [Publication Title: "group

counselling"] OR [Publication Title: "group psychotherapy"] OR [Publication Title: "group psychoeducation"] OR [Publication Title: "group psycho-education"]]

[[Abstract: "ptsd"] OR [Abstract: "posttraumatic stress"] OR [Abstract: "posttraumatic disorder"] OR [Abstract: "posttraumatic symptom"] OR [Abstract: "posttraumatic neuroses"] OR [Abstract: "post-traumatic stress"] OR [Abstract: "post-traumatic disorder"] OR [Abstract: "post-traumatic symptom"] OR [Abstract: "post-traumatic neuroses"] OR [Abstract: "post traumatic stress"] OR [Abstract: "post traumatic disorder"] OR [Abstract: "post traumatic symptom"] OR [Abstract: "post traumatic neuroses"] OR [Abstract: "acute stress disorder"] OR [Abstract: "combat disorder"] OR [Abstract: "war neuroses"]] AND [[Abstract: "group treatment"] OR [Abstract: "group intervention"] OR [Abstract: "group therapy"] OR [Abstract: "group training"] OR [Abstract: "group program"] OR [Abstract: "group session"] OR [Abstract: "group setting"] OR [Abstract: "group factor"] OR [Abstract: "group process"] OR [Abstract: "group counselling"] OR [Abstract: "group psychotherapy"] OR [Abstract: "group psychoeducation"] OR [Abstract: "group psycho-education"]]

Handsearching Journal of Traumatic Stress

The search was conducted three times, focussed on identifying hits from publication titles, abstracts, and keywords as illustrated below.

"(“PTSD” OR “posttraumatic stress” OR “posttraumatic disorder” OR “posttraumatic symptom” OR “posttraumatic neuroses” OR “post-traumatic stress” OR “post-traumatic disorder” OR “post-traumatic symptom” OR “post-traumatic neuroses” OR “post traumatic stress” OR “post traumatic disorder” OR “post traumatic symptom” OR “post traumatic neuroses” OR "acute stress disorder" OR "combat disorder" OR "war neuroses") AND (“group treatment” OR “group intervention” OR “group therapy” OR “group training” OR “group program” OR “group session” OR “group setting” OR “group factor” OR “group process” OR “group counselling” OR “group psychotherapy” OR “group psychoeducation” OR “group psycho-education”)" in **Title** published in "Journal of Traumatic Stress"

"(“PTSD” OR “posttraumatic stress” OR “posttraumatic disorder” OR “posttraumatic symptom” OR “posttraumatic neuroses” OR “post-traumatic stress” OR “post-traumatic disorder” OR “post-traumatic symptom” OR “post-traumatic neuroses” OR “post traumatic stress” OR “post traumatic disorder” OR “post traumatic symptom” OR “post traumatic neuroses” OR "acute stress disorder" OR "combat disorder" OR "war neuroses") AND (“group treatment” OR “group intervention” OR “group therapy” OR “group training” OR “group program” OR “group session” OR “group setting” OR “group factor” OR “group process” OR “group counselling” OR “group psychotherapy” OR “group psychoeducation” OR “group psycho-education”)" in **Keywords** published in "Journal of Traumatic Stress"

"(“PTSD” OR “posttraumatic stress” OR “posttraumatic disorder” OR “posttraumatic symptom” OR “posttraumatic neuroses” OR “post-traumatic stress” OR “post-traumatic disorder” OR “post-traumatic symptom” OR “post-traumatic neuroses” OR “post traumatic

stress” OR “post traumatic disorder” OR “post traumatic symptom” OR “post traumatic neuroses” OR "acute stress disorder" OR "combat disorder" OR "war neuroses") AND (“group treatment” OR “group intervention” OR “group therapy” OR “group training” OR “group program” OR “group session” OR “group setting” OR “group factor” OR “group process” OR “group counselling” OR “group psychotherapy” OR “group psychoeducation” OR “group psycho-education”)" in **Abstract** published in "Journal of Traumatic Stress"

Handsearching Psychological Trauma: Theory, Research, Practice, and Policy

The following search was completed searching across “any field”.

(“PTSD” OR “posttraumatic stress” OR “posttraumatic disorder” OR “posttraumatic symptom” OR “posttraumatic neuroses” OR “post-traumatic stress” OR “post-traumatic disorder” OR “post-traumatic symptom” OR “post-traumatic neuroses” OR “post traumatic stress” OR “post traumatic disorder” OR “post traumatic symptom” OR “post traumatic neuroses” OR "acute stress disorder" OR "combat disorder" OR "war neuroses") AND Any Field: (“group treatment” OR “group intervention” OR “group therapy” OR “group training” OR “group program” OR “group session” OR “group setting” OR “group factor” OR “group process” OR “group counselling” OR “group psychotherapy” OR “group psychoeducation” OR “group psycho-education”) AND Age Group: Adulthood (18 yrs & older)

Government of Canada publications

Searched for “(“PTSD” OR “posttraumatic stress” OR “posttraumatic disorder” OR “posttraumatic symptom” OR “posttraumatic neuroses” OR “post-traumatic stress” OR “post-traumatic disorder” OR “post-traumatic symptom”)

GreyLit.org

We hand searched the following terms and reviewed the hits for inclusion: “PTSD”, “posttraumatic stress”, “posttraumatic disorder”, “posttraumatic symptom”, “posttraumatic neuroses”, “post-traumatic stress”, “post-traumatic disorder”, “post-traumatic symptom”.

NICE.ORG.UK

Hand searched the term “PTSD”.
